# Interspecies Genomic Variation and Transcriptional Activeness of Secondary Metabolism-Related Genes in *Aspergillus* Section *Fumigati*

**DOI:** 10.3389/ffunb.2021.656751

**Published:** 2021-04-16

**Authors:** Hiroki Takahashi, Maiko Umemura, Akihiro Ninomiya, Yoko Kusuya, Masaaki Shimizu, Syun-ichi Urayama, Akira Watanabe, Katsuhiko Kamei, Takashi Yaguchi, Daisuke Hagiwara

**Affiliations:** ^1^Medical Mycology Research Center, Chiba University, Chiba, Japan; ^2^Molecular Chirality Research Center, Chiba University, Chiba, Japan; ^3^Plant Molecular Science Center, Chiba University, Chiba, Japan; ^4^Bioproduction Research Institute, National Institute of Advanced Industrial Science and Technology (AIST), Tsukuba, Japan; ^5^Faculty of Life and Environmental Sciences, University of Tsukuba, Tsukuba, Japan; ^6^Department of Biology, Faculty of Science, Chiba University, Chiba, Japan; ^7^Microbiology Research Center for Sustainability, University of Tsukuba, Tsukuba, Japan

**Keywords:** comparative genomic analysis, *Aspergillus fumigatus*, gliotoxin, terrein, viriditoxin, *Aspergillus* section *Fumigati*, comparative transcriptomic analysis, secondary metabolic gene

## Abstract

Filamentous fungi produce various bioactive compounds that are biosynthesized by sets of proteins encoded in biosynthesis gene clusters (BGCs). For an unknown reason, many BGCs are transcriptionally silent in laboratory conditions, which has hampered the discovery of novel fungal compounds. The transcriptional reactiveness of fungal secondary metabolism is not fully understood. To gain the comprehensive view, we conducted comparative genomic and transcriptomic analyses of nine closely-related species of *Aspergillus* section *Fumigati* (*A. fumigatus, A. fumigatiaffinis, A. novofumigatus, A. thermomutatus, A. viridinutans, A. pseudoviridinutans, A. lentulus, A. udagawae*, and *Neosartorya fischeri*). For expanding our knowledge, we newly sequenced genomes of *A. viridinutans* and *A. pseudoviridinutans*, and reassembled and reannotated the previously released genomes of *A. lentulus* and *A. udagawae*. Between 34 and 84 secondary metabolite (SM) backbone genes were identified in the genomes of these nine respective species, with 8.7–51.2% being unique to the species. A total of 247 SM backbone gene types were identified in the nine fungi. Ten BGCs are shared by all nine species. Transcriptomic analysis using *A. fumigatus, A. lentulus, A. udagawae, A. viridinutans*, and *N. fischeri* was conducted to compare expression levels of all SM backbone genes in four different culture conditions; 32–83% of SM backbone genes in these species were not expressed in the tested conditions, which reconfirmed that large part of fungal SM genes are hard to be expressed. The species-unique SM genes of the five species were expressed with lower frequency (18.8% in total) than the SM genes that are conserved in all five species (56%). These results suggest that the expression tendency of BGCs is correlated with their interspecies distribution pattern. Our findings increase understanding of the evolutionary processes associated with the regulation of fungal secondary metabolism.

## Introduction

Filamentous fungi produce various small molecules known as secondary metabolites (SMs; also called natural products) that are thought to contribute to their survival in environmental niches (Keller, 2015; Künzler, 2018). Fungal SMs are biosynthesized by enzyme sets, which include backbone-producing and tailoring enzymes. The backbone-producing enzymes are represented by non-ribosomal peptide synthetases (NRPSs) and polyketide synthases (PKSs), whereas terpene cyclases and dimethylallyl tryptophan synthases are also involved (Keller, 2019). Genes encoding backbone-producing and tailoring enzymes, transcriptional regulators, and efflux pumps are often arrayed in a biosynthesis gene cluster (BGC). Fungi, including phytopathogens and human pathogens, possess large numbers of SM-producing gene clusters in their genomes, which indicates an ability to produce myriad metabolites that could be used to impact humans (Sanchez et al., 2012; Hansen et al., 2015; Nielsen et al., 2017; Vesth et al., 2018; Kjærbølling et al., 2020).

Fungal SM gene clusters are, in general, transcriptionally silent in tested lab conditions, which makes it difficult for us to comprehensively explore fungal SMs and to understand the ecological roles of the SMs (Brakhage and Schroeckh, 2011). For example, genomic study revealed that >30 genes encoding enzymes to produce SM backbones were found in *Aspergillus fumigatus, A. niger*, and *A. oryzae*, 74.2–91.4% of which were not expressed or were expressed at a very low level in any of the cell types (hyphae, resting conidia, or germinating conidia; Hagiwara et al., 2016). One explanation for the low expression of SM-related genes in laboratory-controlled conditions is that unknown ecological cues trigger fungal SM production but cannot be reproduced in the laboratory. Many researchers have attempted to artificially activate such silent SM genes by coculturing multiple microorganisms (Netzker et al., 2018), adding inhibitors for the histone deacetylases that regulate epigenetic status (Pfannenstiel and Keller, 2019), or treatment with plant hormones (Morishita et al., 2019). The molecular mechanisms underlying artificial activation of SM-related genes remain to be investigated.

Recent progress in DNA sequencing technology has advanced our understanding of fungal SM-related gene distribution across species. Comparative genomics has shown that SM-related genes (BGCs) are species-unique or narrowly taxonomically distributed. For example, among four representatives of *Aspergillus* fungi—*A. fumigatus, A. nidulans, A. niger*, and *A. oryzae—*no BGCs are shared by all species, and 91.6–96.1% of the BGCs are species-unique (Lind et al., 2015). This is in sharp contrast to primary metabolic genes, where only 7.5–15.4% of the genes are species-unique. However, when focusing on more closely related species that belong to same section, a taxonomic group below genus but above species, several BGCs are shared in common among species of *Aspergillus* section *Nigri* or *Aspergillus* section *Flavi* (Vesth et al., 2018; Kjærbølling et al., 2020). These reports provide an evolutionary insight into how secondary metabolic pathways evolve and degenerate in filamentous fungi.

*A. fumigatus* is a life-threatening pathogenic fungus of humans and is a representative member of *Aspergillus* section *Fumigati* (Rokas et al., 2020). This group also includes nonpathogenic fungi, such as *Neosartorya fischeri*, which has been well-studied by comparison with *A. fumigatus* in terms of genome structure, pathogenicity, drug resistance, toxin production, and SMs (Fedorova et al., 2008; Mead et al., 2019; Knowles et al., 2020, Steenwyk et al., 2020). Only a few extrolites of *A. fumigatus* and *N. fischeri* are overlapped even though 30.3% of *A. fumigatus* SM-related genes are also found in *N. fischeri* (Mead et al., 2019), which suggests that transcriptional or/and translational regulation of the SM genes are different in each species. Similarly, *A. novofumigatus*, which also belongs to *Aspergillus* section *Fumigati*, was reported to share 70.5% of *A. fumigatus* SM-related genes (Kjærbølling et al., 2018), and few metabolites were reported to be shared with *A. fumigatus*.

In the present study, we compared the SM-related genes among closely-related fungal species of *Aspergillus* section *Fumigati* to determine how many SM genes are shared or unique among this group of species. Transcriptome analysis was then performed for five species to gain an overview of how many of the SM-related genes are transcriptionally silent, and insight into the relationships between the tendency for expression and the interspecies distribution of SM genes. The expression profiles of the common BGCs were diverse among the species, whereas species-unique SM-related genes were frequently not expressed in the tested culture conditions.

## Results

### Sequencing and Updating Genomes of Species in *Aspergillus* Section *Fumigati*

To compare more genomes of species in *Aspergillus* section *Fumigati, A. viridinutans* IFM 47045 and *A. pseudoviridinutans* IFM 55266 were newly sequenced using the Illumina short read system. The sequenced reads were assembled into 47 and 24 scaffolds, with total length 34.88 and 33.33 Mb, respectively (Table 1). The numbers of predicted proteins were 10,039 and 11,281, and the quality of the genome assembly was confirmed (presence of BUSCO genes: 99.7 and 99.6%), respectively. Our group has reported genome sequences of *A. lentulus* IFM 54703 and *A. udagawae* IFM 46973 (Kusuya et al., 2015, 2016). To improve the genome datasets, we here reassembled the sequence of *A. udagawae* IFM 46973 using ALLPATHS-LG (ver. R52488) and reannotated the genes of *A. lentulus* IFM 54703 and *A. udagawae* IFM 46973 (the updated gene IDs are designated Alt_000001-T1 and Aud_000001-T1, respectively). As a result, the sequence assembly was much improved; the genomes of both species were composed of 17 scaffolds. The four abovementioned genome datasets were deposited and updated in the NCBI (https://www.ncbi.nlm.nih.gov/).

**Table 1:** Genome characteristics and assembly of *Aspergillus* section *Fumigati* strains.

**Strains**	**Genome size [Mb]**	**# of scaffolds**	**Average [kb]**	**N50 [kb]**	**# of Predicted proteins**	**# of Species-unique genes**	**BUSCO (4.0.6) eurotiales_odb10_genome^a^**	**BUSCO (4.0.6) eurotiales_odb10_protein^a^**	**References**
*A. fumigatus* Af293	29.42	9	3268.9	3948.4	9,841	1,507	C:99.7%[S:99.4%,D:0.3%],F:0.0%,M:0.3%	C:98.5%[S:98.2%,D:0.3%],F:0.7%,M:0.8%	Nierman et al., 2005
*N. fischeri* NRRL 181^T^	32.55	976	33.3	2929.1	10,406	1,245	C:99.7%[S:99.2%,D:0.5%],F:0.0%,M:0.3%	C:98.3%[S:98.0%,D:0.3%],F:1.3%,M:0.4%	Fedorova et al., 2008
*A. lentulus* IFM 54703^T^	30.77	12	2564.5	4166.7	10,319	948	C:99.7%[S:99.5%,D:0.2%],F:0.0%,M:0.3%	C:98.8%[S:98.7%,D:0.1%],F:0.6%,M:0.6%	Kusuya et al., 2016
*A. udagawae* IFM 46973^T^	32.25	17	1897.1	4123.3	10,796	1,420	C:99.7%[S:99.5%,D:0.2%],F:0.0%,M:0.3%	C:99.1%[S:98.9%,D:0.2%],F:0.2%,M:0.7%	Kusuya et al., 2015
*A. viridinutans* IFM 47045^T^	34.88	47	742.3	2861.4	10,039	1,259	C:99.7%[S:99.5%,D:0.2%],F:0.0%,M:0.3%	C:99.2%[S:99.0%,D:0.2%],F:0.2%,M:0.6%	This study
*A. pseudviridinutans* IFM 55266	33.33	24	1388.7	4753.4	11,281	1,779	C:99.6%[S:99.4%,D:0.2%],F:0.0%,M:0.4%	C:99.1%[S:98.9%,D:0.2%],F:0.2%,M:0.7%	This study
*A. fumigatiaffinis* CNM-CM6805	33.47	1,055	31.7	161.1	10,468	1,717	C:98.7%[S:98.4%,D:0.3%],F:0.5%,M:0.8%	C:96.1%[S:95.8%,D:0.3%],F:1.2%,M:2.7%	Dos Santos et al., 2020
*A. novofumigatus* IBT 16806	32.44	62	523.2	3768.3	11,534	2,618	C:97.8%[S:97.6%,D:0.2%],F:0.2%,M:2.0%	C:97.9%[S:97.5%,D:0.4%],F:0.9%,M:1.2%	Kjærbølling et al., 2018
*A. thermomutatus* HMR AF 39	30.94	647	47.8	93.3	9,702	2,396	C:98.8%[S:98.6%,D:0.2%],F:0.5%,M:0.7%	C:94.0%[S:93.8%,D:0.2%],F:1.1%,M:4.9%	Parent-Michaud et al., 2019

a *C, S, D, F, and M indicate complete BUSCOs, complete single-copy BUSCOs, complete duplicated BUSCOs, fragmented BUSCOs, and missing BUSCOs, respectively*.

**Figure 1:**
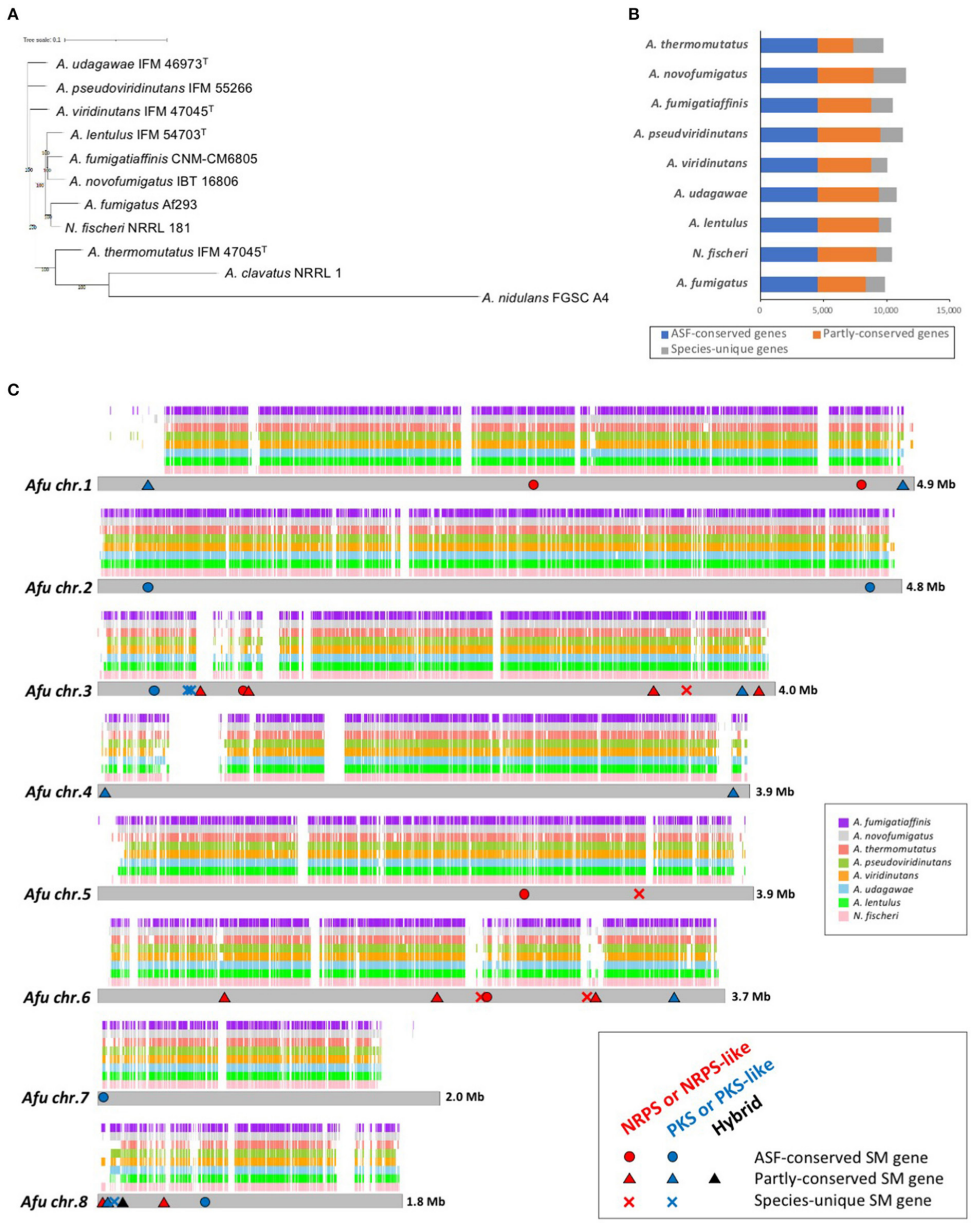
Genome characteristics of *Aspergillus* section *Fumigati* strains. **(A)** Phylogenetic tree of the nine species of *Aspergillus* section *Fumigati* used here in addition to *A. clavatus* and *A. nidulans* as the outgroup. The tree was constructed using iTOL. **(B)** The numbers of genes that are conserved across the section (“ASF-conserved genes”), partly conserved, or species-unique are shown. **(C)** Whole-genome synteny plot based on *A. fumigatus* chromosomes. The syntenic genes are mapped to *A. fumigatus* chromosomes. The positions of the ASF-conserved, partly-conserved, and species-unique SM backbone genes of *A. fumigatus* are indicated with circles, triangles, and crosses, respectively. Genes encoding non-ribosomal peptide synthetase (NRPS) or NRPS-like proteins are colored red, polyketide synthase (PKS) or PKS-like proteins in blue, and PKS-NRPS hybrid proteins in black.

In addition to these genomes, genome information for *A. fumigatus* Af293 (Nierman et al., 2005), *N. fischeri* NRRL 181 (Fedorova et al., 2008), *A. fumigatiaffinis* CNM-CM6805 (Dos Santos et al., 2020), *A. novofumigatus* IBT 16806 (Kjærbølling et al., 2018), and *A. thermomutatus* HMR AF 39 (Parent-Michaud et al., 2019) was available at the NCBI and was retrieved for this study. Consequently, a phylogenetic tree was constructed using conserved genes in these nine closely-related species in *Aspergillus* section *Fumigati*. *N. fischeri* was the closest relative to *A. fumigatus* (Figure 1A).

The numbers of predicted proteins in the fungi ranged from 9,702 to 11,534 and are summarized in Table 1. By reciprocal best hit (RBH) search with strict criteria (>80% identity and >80% of length covered), 4,493 genes were determined to be orthologs conserved in the nine *Aspergillus* section *Fumigati* species (referred to hereafter as “ASF-conserved genes”), which represented the core-genome (Figure 1B). Among the nine species, there were 948–2,618 species-unique genes (i.e., having no orthologs in other species) (Table 1; Figure 1B).

Synteny analysis of the genomes revealed that most of the *A. fumigatus* genome is covered by the other species, but that there are several regions unique to *A. fumigatus* (Figure 1C). The number of *A. fumigatus* genes that reside in the syntenic region to each other species was 6,874 (*A. thermomutatus*) to 8,266 (*N. fischeri*) (69.9–84.0% of *A. fumigatu*s genes). This further suggested that *N. fischeri* is the closest relative to *A. fumigatu*s among the *Aspergillus* section *Fumigati* species.

### Comparative Genomics Regarding SM-Related Genes

**Figure 2:**
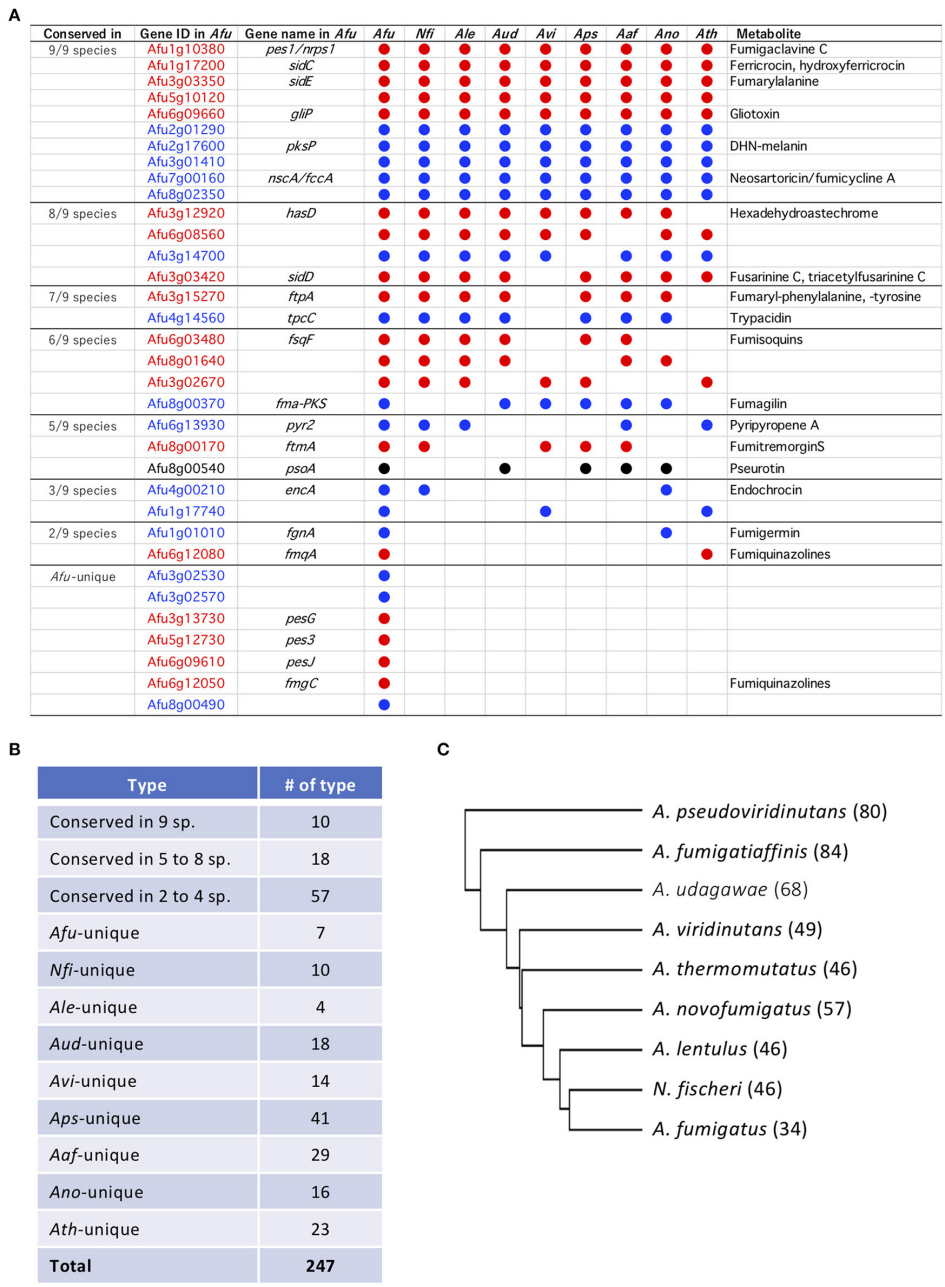
Secondary metabolite (SM) backbone genes conserved across *Aspergillus* section *Fumigati*. **(A)** Summary of *A. fumigatus* SM backbone genes. Some are conserved in other *Fumigati* species. *Afu, A. fumigatus; Nfi, Neosartorya fischeri; Ale, A. lentulus; Aud, A. udagawae; Avi, A. viridinutans; Aps, A. pseudoviridinutans; Aaf, A. fumigatiaffinis; Ano, A. novofumigatus; Ath, A. thermomutatus*. **(B)** Summary of the numbers of SM gene types. **(C)** A cladogram was constructed using a binary matrix (presence/absence of the PKSs and NRPSs) with Cluster 3.0. The tree was constructed and drawn using Tree View. The numbers of SM backbone genes are shown in parentheses behind the species names.

SM backbone genes encoding PKSs, NRPSs, and PKS-NRPS hybrids were identified from the genome data using antiSMASH software with manual refinement. In total, 34–84 such genes were identified in the nine species (Table 2; Supplementary Table 1). Orthologous SM-related proteins that showed identities of >80% in >80% of the protein were identified. Twenty-seven of the 34 SM backbone genes in *A. fumigatus* have orthologs in other species, and seven genes are unique to *A. fumigatus* (Figure 2A). The other species share 17–22 of the SM backbone genes with *A. fumigatus*. *N. fischeri* had the most SM genes in common with *A. fumigatus* (Figure 2A). Notably, 10 genes were shared among all nine studied species of *Aspergillus* section *Fumigati* (referred to hereafter as “ASF-conserved SM genes”) (Figure 2B). Meanwhile, there were 18 and 57 genes that were conserved in 5–8 species and 2–4 species, respectively. The species-unique SM backbone genes were identified in each species. *A. lentulus* has 4 (the fewest), and *A. pseudoviridinutans* has 41 species-unique SM backbone genes (the most). In total, 510 SM backbone genes were identified and grouped into 247 orthologous types (Figure 2B). A cladogram was generated based on a binary matrix (presence/absence of the SM backbone genes), which revealed that *N. fischeri* is the most closely related species to *A. fumigatus* based on the SM backbone protein distribution across species (Figure 2C).

**Table 2:** The numbers of SM backbone genes predicted in the genome of strains of *Aspergillus* section *Fumigati*.

**Strains**	**NRPS or NRPS-like**	**PKS or PKS-like**	**Hybrid**	**Total**
*A. fumigatus* Af293	18	15	1	34
*N. fischeri* NRRL 181^T^	28	17	1	46
*A. lentulus* IFM 54703^T^	22	20	4	46
*A. udagawae* IFM 46973^T^	30	35	3	68
*A. viridinutans* IFM 47045^T^	21	26	2	49
*A. pseudviridinutans* IFM 55266	39	36	5	80
*A. thermomutatus* HMR AF 39	48	31	5	84
*A. novofumigatus* IBT 16806	24	28	5	57
*A. fumigatiaffinis* CNM-CM6805	22	20	4	46

### Characterization of the ASF-Conserved SM Gene Clusters

The 10 ASF-conserved SM backbone genes include five NRPSs and five PKSs, among which six were previously characterized in *A. fumigatus* as being involved in the biosynthesis of fumigaclavine C (O'Hanlon et al., 2012), ferricrocin (Schrettl et al., 2007), fumarylalanine (Steinchen et al., 2013), gliotoxin (Cramer et al., 2006), 1,8-dihydroxynaphthalene (DHN)-melanin (Langfelder et al., 1998), and neosartoricin/fumicyclines (Chooi et al., 2013; König et al., 2013) (Figure 2A). The BGCs containing the ASF-conserved SM backbone genes (hereafter designated BGC1 to BGC10) were compared among the nine *Aspergillus* section *Fumigati* species (Supplementary Table 2). BGC1 and BGC3 were almost perfectly conserved across the species in terms of gene composition, gene order, and the similarity of the encoded proteins (Supplementary Figure 1). Although there were a few examples of gene loss or low protein similarity, BGC2, BGC4, BGC5, BGC8, and BGC9 were also well-conserved among the species. Meanwhile, BGC6, BGC7, and BGC10 were diverse due to extensive gene loss and low protein similarity in several components. In particular, BGC7 of *A. fumigatus* was unique because four of the 10 component genes have no orthologs in the other species (Supplementary Figure 1). BGC2, BGC7, BGC8, and BGC9 contain one or two genes encoding a transcription factor (TF), which are conserved in all nine species (Supplementary Table 2; Supplementary Figure 1). Interestingly, most of the TFs are located at the ends of gene clusters.

### Comparative Transcriptome Analysis of SM Backbone Genes

**Figure 3:**
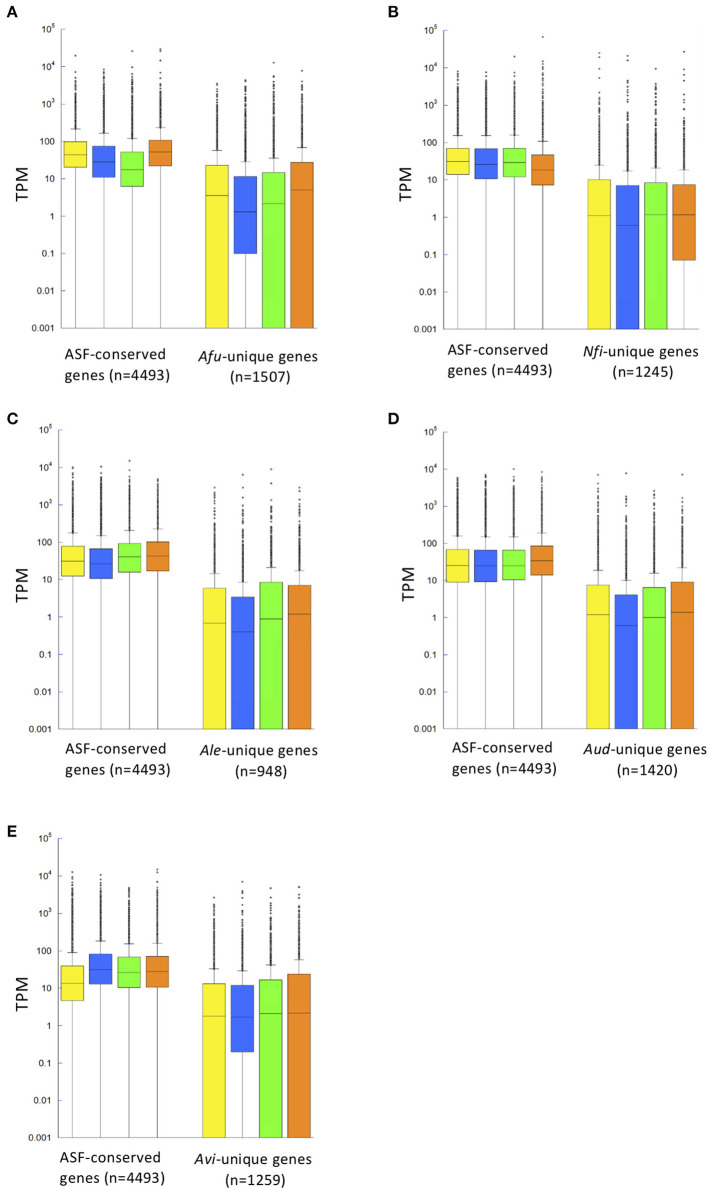
Distribution of gene expression levels shown in a box plot. Gene expression patterns of ASF-conserved genes as the core genome (*n* = 4,493) and species-unique genes are shown for *A. fumigatus* **(A)**, *N. fischeri* **(B)**, *A. lentulus* **(C)**, *A. udagawae* **(D)**, and *A. viridinutans* **(E)** in four different conditions [potato dextrose broth (PDB), Czapek-Dox medium (CD), Sabouraud broth (SB), and potato dextrose agar (PDA)], indicated by yellow, blue, green, and orange boxes, respectively. Gene expression levels are described in Transcripts Per Kilobase Millions (TPM) obtained in RNA-sequencing analysis. The box plot graph is drawn using PlotsOfData (https://huygens.science.uva.nl/PlotsOfData/) (Postma and Goedhart, 2019).

To gain insights into SM gene expression, transcriptomic analysis was conducted using five representative species of *Aspergillus* section *Fumigati*: *A. fumigatus, N. fischeri, A. lentulus, A. udagawae*, and *A. viridinutans*. The fungal strains were cultivated in four different media: potato dextrose broth (PDB), Czapek-Dox medium (CD), Sabouraud broth (SB), and potato dextrose agar (PDA). In *A. fumigatus*, the median numbers of Transcripts Per Kilobase Millions (TPMs) of ASF-conserved genes (*n* = 4,493) were 44.6, 28.3, 17.5, and 52.9 for culture on PDB, CD, SB, and PDA, respectively, much higher than the values for *A. fumigatus*-unique genes (3.6, 1.5, 2.2, and 5.0; *n* = 1,507) (Figure 3A). This was also the case for the other species (Figures 3B–E). These data indicated that a set of genes that was well-conserved among aspergilli was transcriptionally more active than species-unique genes in all the tested species.

**Figure 4:**
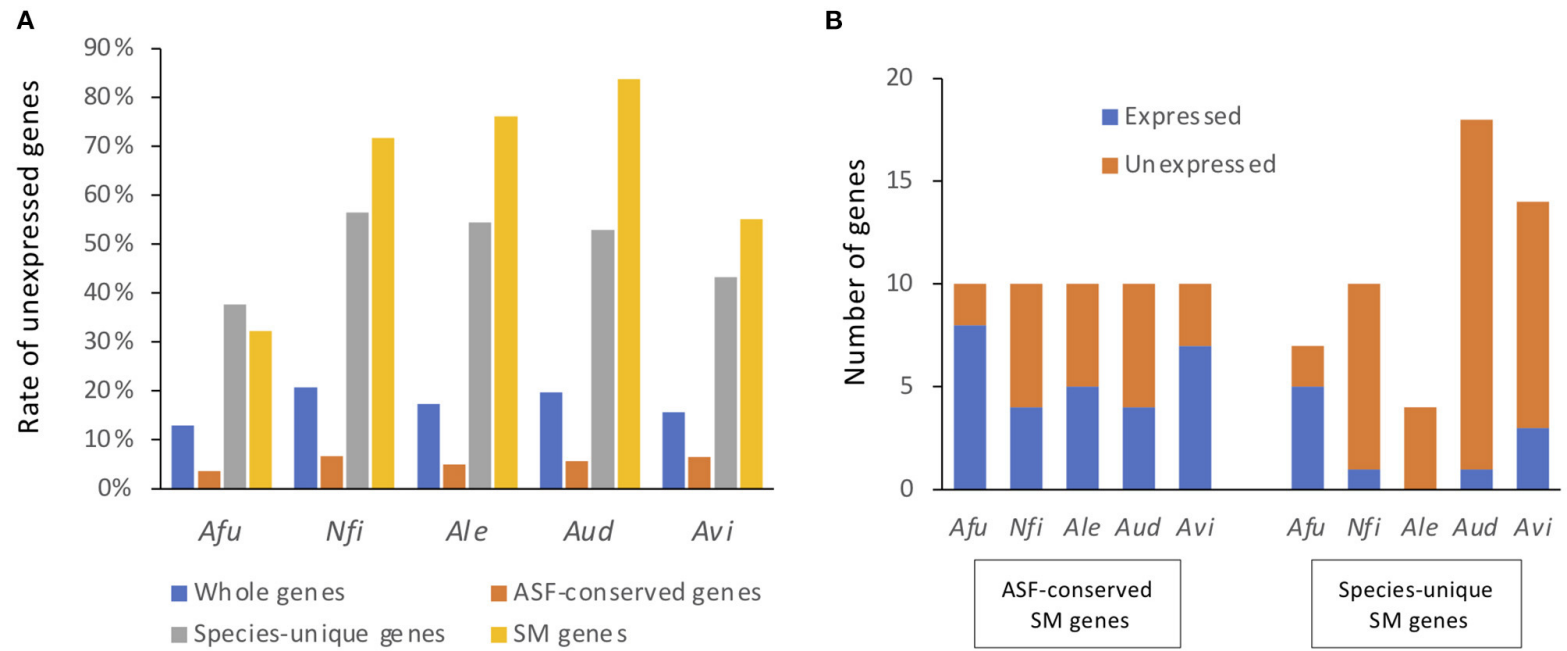
Proportions and numbers of unexpressed SM backbone genes. Genes with TPMs > 5% of the mean TPM in either condition were regarded as expressed genes, whereas the remainder were regarded as unexpressed genes. **(A)** The proportion of genes that were not expressed in any culture condition are shown for all genes, ASF-conserved genes, species-unique genes, and SM backbone genes. *Afu, A. fumigatus; Nfi, N. fischeri; Ale, A. lentulus; Aud, A. udagawae; Avi, A. viridinutans*. **(B)** The numbers of SM backbone genes that were expressed and not expressed in any culture condition.

When a gene with an expression level >^1^/${\;}_{20}^{\text{th}}$ of the mean TPM was considered as being expressed, 12.8% of *A. fumigatus* genes were not expressed in any of the conditions tested (Figure 4A), and were thus considered to be silent genes. Only 3.5% of the ASF-conserved genes were silent in *A. fumigatus*. In contrast, 37.7% of species-unique genes were silent. This tendency was also observed in the other species (Figure 4A). With regard to SM backbone genes, 32.3% of the genes were silent in *A. fumigatus*, and the proportion of silent SM backbone genes was 71.7, 76.0, 83.8, and 55.1% in *N. fischeri, A. lentulus, A. udagawae*, and *A. viridinutans*, respectively (Figure 4A). These data highlight that the proportion of SM backbone genes that were expressed was quite low in species of *Aspergillus* section *Fumigati*.

For ASF-conserved SM genes, the proportion of silent genes ranged from 20 to 60% in the species of *Aspergillus* section *Fumigati* (44% in total) (Figure 4B). In contrast, 28.5–100% of the species-unique SM genes were not expressed in any of the culture conditions tested (81.1% in total) (Figure 4B). These results suggest that the ASF-conserved SM genes are transcriptionally more active than the less conserved SM genes. Lists of SM backbone genes with expression values are shown in Supplementary Tables 3–7.

### Gene Cluster Identification by Transcriptional Dataset Using MIDDAS-M

The SM gene clusters that were activated in (a) specific condition(s) were further analyzed. We sought gene clusters whose component genes were coordinately regulated using the MIDDAS-M program (Umemura et al., 2013). Consequently, 14, 13, 12, 12, and 19 sets of genes were found to be expressed as clustered genes in *A. fumigatus, N. fischeri, A. lentulus, A. udagawae*, and *A. viridinutans*, respectively (Supplementary Table 8). Of these, 4, 6, 3, 5, and 9 expressed clusters contained SM backbone genes, respectively (Table 3). All these clusters belonged to the ASF-conserved or partly conserved SM genes, and no cluster with species-unique SM genes was found by the MIDDAS-M analysis. Interestingly, most of the expressed clusters have been characterized and are predicted to produce known metabolites. The gliotoxin (*gliP*) cluster was expressed in *A. fumigatus, A. lentulus*, and *A. viridinutans*, and the DHN-melanin (*pksP*) cluster in *A. fumigatus* and *A. viridinutans*. Furthermore, the fusarinine C (*sidD*), ferricrocin (*sidC*), hexadehydroastechrome (*hasD*), and fumitremorgin (*ftmA*) clusters was expressed in multiple species studied here. In addition to the metabolites whose BGCs are not present in *A. fumigatus*, the clusters for terrein and viriditoxin were coordinately expressed in *A. lentulus* and *A. viridinutans*, respectively.

**Table 3:** Coordinately expressed SM gene clusters identified by MIDDAS analysis.

**Strain**	**Cluster start**	**Cluster end**	**# of genes**	**SM backbone gene in the cluster**	**Predicted metabolite**	**# of conserved species**
*A. fumigatus*	Afu2g17515	Afu2g17600	8	Afu2g17600 (*pksP*)	DHN-melanin	9 (BGC4)
	Afu4g14460	Afu4g14580	13	Afu4g14560 (*tpcC*)	Trypacidin	Partry-conserved: 7
	Afu6g09600	Afu6g09745	16	Afu6g09610, Afu6g09660 (*gliP*)	Gliotoxin	9 (BGC8)
	Afu8g00370	Afu8g00580	21	Afu8g00370 (*fmaB*), Afu8g00490, Afu8g00540 (*psoA*)	Fmagilin, pseurotin	Partry-conserved: 6, 5, 1
*N. fischeri*	NFIA_005560	NFIA_005620	7	NFIA_005590 (*sidD*)	Fusarinine C, triacetylfusarinine C	Partry-conserved: 8
	NFIA_008100	NFIA_008190	10	NFIA_008170 (*sidC*)	Ferricrocin	9 (BGC2)
	NFIA_062240	NFIA_062290	6	NFIA_062250		Partry-conserved: 2
	NFIA_064390	NFIA_064420	4	NFIA_064400 (*hasD*)	Hexadehydroastechrome	Partry-conserved: 8
	NFIA_093690	NFIA_093740	6	NFIA_093690 (*ftmA*)	Fumitremorgins	Partry-conserved: 5
	NFIA_100450	NFIA_100520	8	NFIA_100520 (*pyr2*)	Pyripyropene A	Partry-conserved: 5
*A. lentulus*	Alt_003617-T1	Alt_003624-T1	8	Alt_003621-T1 (*hasD*)	Hexadehydroastechrome	Partry-conserved: 8
	Alt_006925-T1	Alt_006934-T1	10	Alt_006934-T1 (*gliP*)	Gliotoxin	9 (BGC8)
	Alt_007753-T1	Alt_007763-T1	11	Alt_007763-T1 (*terA*), Alt_007762-T1 (*terB*)	Terrein	Partry-conserved: 3
*A. udagawae*	Aud_002752-T1	Aud_002766-T1	14	Aud_002754-T1 (*sidE*), Aud_002763-T1 (*sidD*)	Fumarylalanine, fusarinine C, triacetylfusarinine C	9 (BGC6)
	Aud_003574-T1	Aud_003581-T1	8	Aud_003577-T1 (*hasD*)	Hexadehydroastechrome	Partry-conserved: 8
	Aud_004514-T1	Aud_004525-T1	12	Aud_004523-T1 (*gliP*)	Gliotoxin	9 (BGC8)
	Aud_008852-T1	Aud_008859-T1	8	Aud_008854-T1		Partry-conserved: 3
	Aud_009320-T1	Aud_009344-T1	25	Aud_009335-T1		Partry-conserved: 2
*A. viridinutans*	Avi_000056-T1	Avi_000060-T1	5	Avi_000055-T1, (*pksP*)	DHN-melanin	9 (BGC4)
	Avi_003609-T1	Avi_003616-T1	8	Avi_003616-T1 (*vdtA*)	Viriditoxin	Partry-conserved: 3
	Avi_003634-T1	Avi_003641-T1	8	Avi_003638-T1		Partry-conserved: 2
	Avi_005751-T1	Avi_005756-T1	6	Avi_005753-T1 (*hasD*)	Hexadehydroastechrome	Partry-conserved: 8
	Avi_005973-T1	Avi_005983-T1	11	Avi_005973-T1		Partry-conserved: 3
	Avi_007490-T1	Avi_007496-T1	7	Avi_007494-T1		Partry-conserved: 2
	Avi_009093-T1	Avi_009095-T1	3	Avi_009095-T1 (*sidC*)	Ferricrocin	9 (BGC2)
	Avi_002834-T1	Avi_002841-T1	8	Avi_002834-T1 (*ftmA*)	Fumitremorgin	Partry-conserved: 5
	Avi_003350-T1	Avi_003368-T1	18	Avi_003364-T1 (*nscA/fccA*), Avi_003357-T1	Neosartoricin/fumicycline A	9 (BGC9)

**Figure 5:**
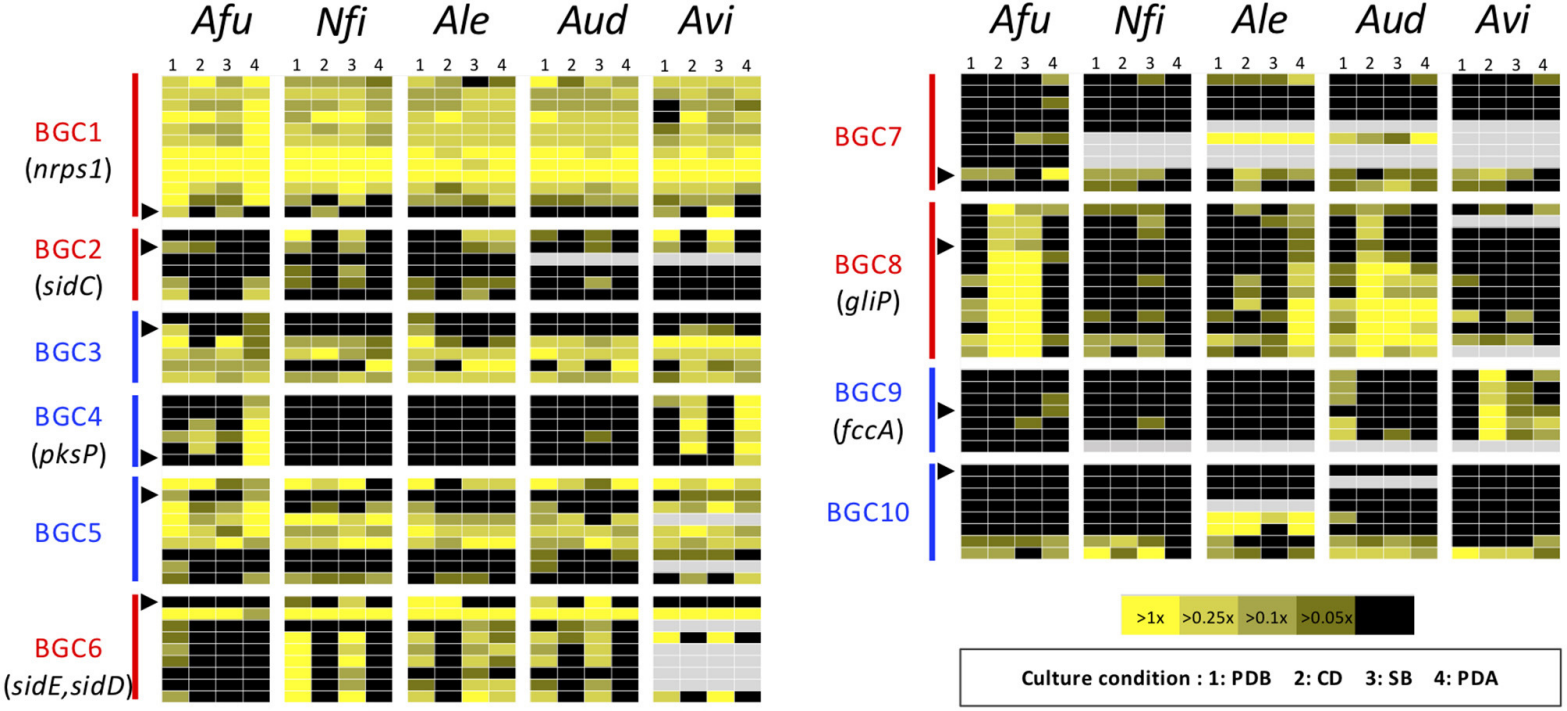
Heat map revealing expression profiles of the genes in ASF-conserved biosynthesis gene clusters. The color of the bar between the biosynthesis gene cluster (BGC) ID and the panels indicates the type of SM backbone gene, as follows: red: NRPS or NRPS-like; blue: PKS or PKS-like. A black triangle indicates the SM backbone gene in the BGC. Gray panels indicate the absence of the corresponding gene. *Afu, A. fumigatus; Nfi, N. fischeri; Alu, A. lentulus; Aud, A. udagawae; Avi, A. viridinutans*.

To gain more insight into variations in the expression patterns of the clusters across the species, expression levels of the component genes in BGCs were depicted using a heat map (Figure 5). This confirmed the result from MIDDAS-M analysis that BGC4 (for *pksP*) was exclusively expressed in PDA in *A. fumigatus* and *A. viridinutans*, and that BGC8 (for *gliP*) was expressed in *A. fumigatus* in CD and SB, in *A. lentulus* in PDA, and in *A. udagawae* in CD. BGC1 was transcriptionally active in a cluster-wide manner in all conditions in all species.

### Characterization of Gliotoxin, Terrein, and Viriditoxin Clusters

The transcriptome data revealed coordinate expression of some BGCs, suggesting productions of some metabolites, which have not been reported in the species. First, we examined whether *A. lentulus* and *A. udagawae* are capable of producing gliotoxin in CD. No production of gliotoxin was detectable in these species, although *A. fumigatus* produced the metabolite (Supplementary Figure 2).

**Figure 6:**
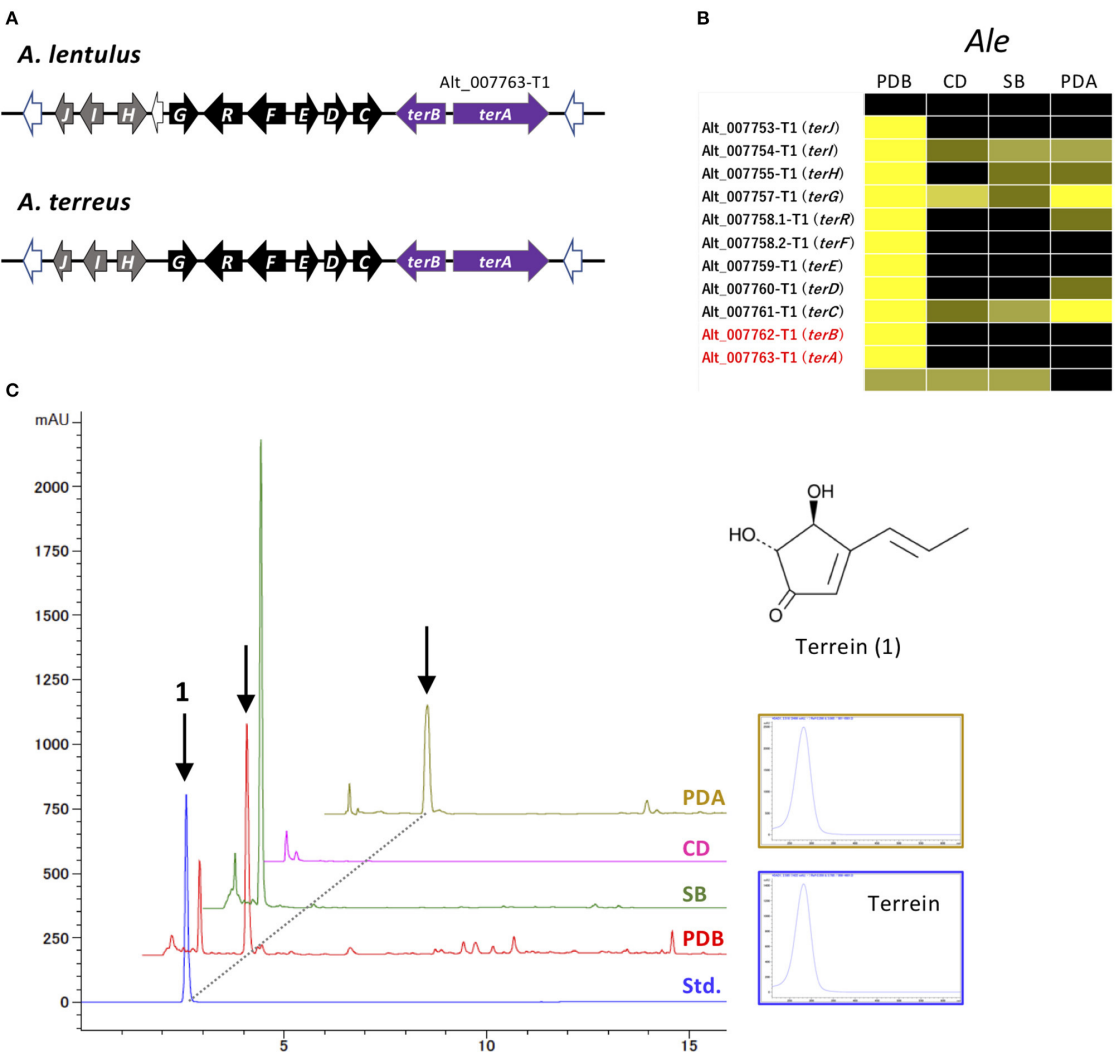
Characterization of the *ter* cluster and terrein production in *A. lentulus*. **(A)** Schematic structure of the *ter* cluster in *A. lentulus* and *A. terreus*. The SM backbone genes (PKSs) are indicated by purple arrows. Genes (*terG, terH, terI*, and *terJ*) whose involvement in terrein production remains obscure in *A. terreus* are indicated by gray arrows. **(B)** The expression profiles of *ter* genes in *A. lentulus* shown using a heat map. **(C)** The production of terrein in *A. lentulus*. The strains were cultivated in PDB, SB, CD, and PDA, and ethyl acetate-derived culture extracts were analyzed using high-performance liquid chromatography. Terrein (**1**) production was identified by comparison with the standard compound (Std.). The corresponding peaks are indicated by arrows, and the UV spectrum from a PDA culture is compared with that of the standard compound.

The MIDDAS-M analysis also highlighted that the cluster for terrein, containing two PKSs, Alt_007763-T1 and Alt_007763-T2, was markedly activated in *A. lentulus*. This cluster is very similar to the *ter* cluster of *A. terreus* in terms of gene content, order, and directions (Figure 6A; Supplementary Table 9) (Zaehle et al., 2014). The cluster in *A. lentulus* was coordinately expressed, with the highest expression level observed in PDB (Figure 6B). Culture extracts were examined by comparison with the standard compound terrein, revealing that *A. lentulus* produced terrein in PDB and on PDA (Figure 6C).

**Figure 7:**
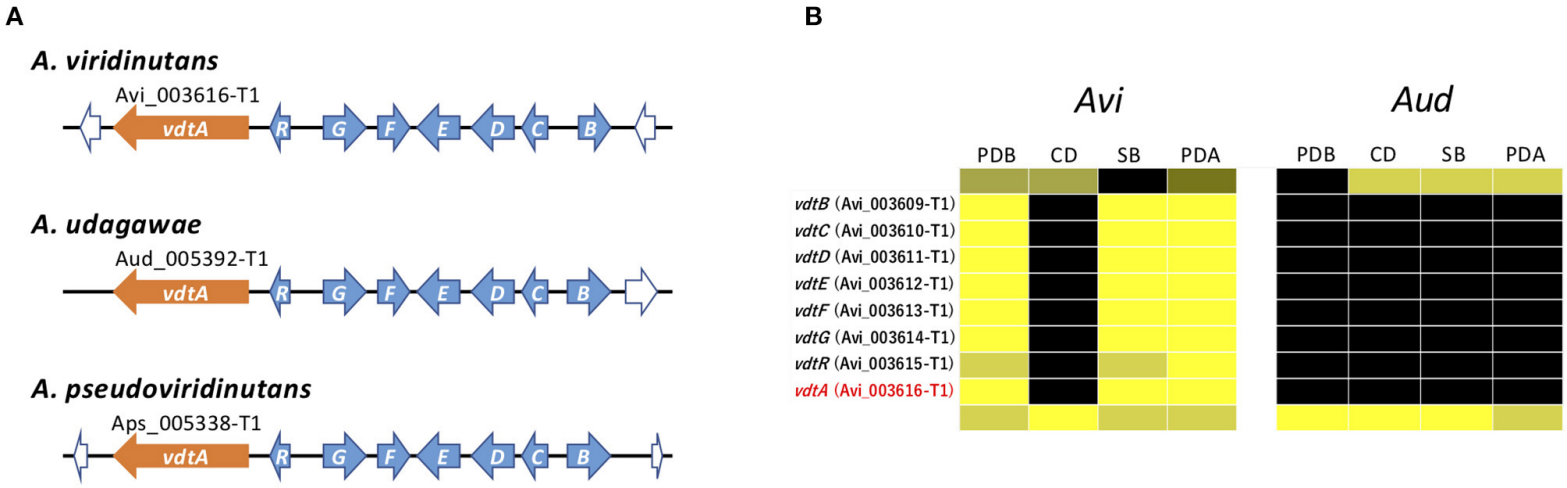
Characterization of *vdt* cluster. **(A)** Schematic structure of the *vdt* cluster from *A. viridinutans, A. udagawae*, and *A. pseudoviridinutans*. The SM backbone genes (PKSs) are indicated by orange arrows. The other component genes are indicated by light blue arrows. **(B)** The expression profiles of *vdt* genes in *A. viridinutans* and *A. udagawae* shown using a heat map.

The *vdt* cluster for viriditoxin, which contains eight genes, has been reported in *A. viridinutans* (Urquhart et al., 2019) (Figure 7A). In *A. viridinutans*, the cluster was highly expressed in a coordinated manner in PDB, SB, and PDA (Figure 7B). On the basis of genome comparison, this cluster is also present in *A. udagawae* and *A. pseudoviridinutans*, and it was found that the genetic content of the clusters is well-conserved between these species (Figure 7A). It was of interest that no genes of the *vdt* cluster were expressed in *A. udagawae* in any of the studied conditions (Figure 7B). This suggested that the *vdt* cluster was active in *A. viridinutans*, but silent in *A. udagawae*.

### Comparative *in silico cis*-Element Analysis of the *gli, ter*, and *vdt* Clusters

**Figure 8:**
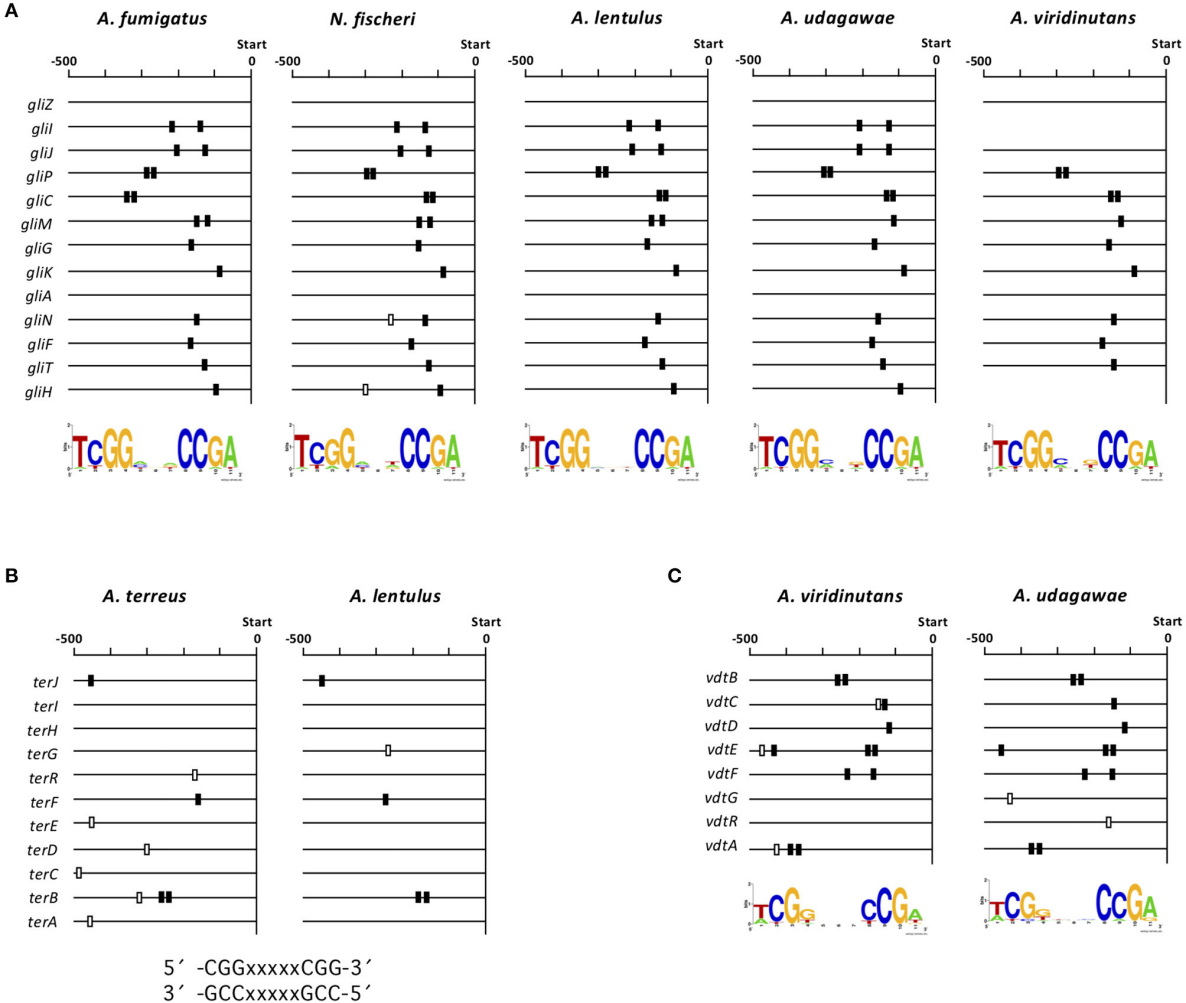
Consensus sequences in the promoter regions of *gli, ter*, and *vdt* genes. **(A)** The consensus sequences were detected in the 13 *gli* genes in each species using BioProspector (Release 2), and the top hit motifs are shown under the map. The position of the sequence (TCGGNNNCCGA) is indicated by a box. The black boxes indicate consensus sequences that were considered to correspond to those in the other species tested. **(B)** Consensus sequences were sought in the 11 *ter* genes in *A. lentulus* and *A. terreus* by manual inspection. The positions of the reported consensus sequences (CCGNNNNNCCG and CGGNNNNNCGG) in the cluster is indicated by black or white boxes. **(C)** Consensus sequences were detected in the eight *vdt* genes in *A. viridinutans* and *A. udagawae* using BioProspector (Release 2), and the top hit motifs are shown under the map. Positions of the sequence (TCGGNNNCCGA) are indicated by black or white boxes. −500, 500-bp upstream from the translational initiation site of each gene.

To gain deeper insight into interspecies variations in the transcriptional regulation of the clusters for gliotoxin, terrein, and viriditoxin biosynthesis, we investigated whether there are *cis*-elements in the promoter regions of each component gene in each cluster. Each cluster contains a gene encoding a C6-type transcription factor (GliZ, TerR, and VdtR) that may function as a cluster-specific transcriptional regulator (Supplementary Figure 1H, Figures 6A, 7A). Because C6-type transcription factors reportedly bind to inverted CGG triplets spaced by several nucleotides, such as CGG(N_x_)CCG (MacPherson et al., 2006), we sought to identify such bipartite motifs conserved in the promoter regions of component genes in the *gli, ter*, and *vdt* clusters using BioProspector (Liu et al., 2001). As binding site 5′-TCGG(N_3_)CCGA-3′ has been reported for the cluster-specific transcription factor GliZ (Schoberle et al., 2014), a palindromic sequence was sought in the *gli* clusters of *A. fumigatus, N. fischeri, A. lentulus, A. udagawae*, and *A. viridinutans*, setting the gap as three bases (Figure 8A). The consensus sequence was found to be highly conserved among the five closely-related *Aspergillus* section *Fumigati* species in terms of position in the promoter region of each gene. No consensus sequence was detected for the *gliZ* gene in any of the five species; this has been reported previously for *A. fumigatus* (Schoberle et al., 2014).

For the *ter* cluster of *A. terreus*, the consensus sequence for binding of TerR, a cluster specific transcription factor, was proposed to be 5′-TCGGHHWYHCGG-3′ (Gressler et al., 2015). Thus, similar sequences (5′-CGGXXXXXCGG-3′ and 5′-CCGXXXXXCCG-3′) were sought in the *ter* cluster of *A. lentulus*. Four *ter* genes contained either of the sequences in the promoter region, and four of the five sequences corresponded to those in *A. terreus* (Figure 8B). No consensus sequence has been reported for the *vdt* cluster in any fungal species. Thus, we sought such a sequence using several combinations of parameters. When the gap was set as three bases, the well-conserved sequence 5′-TCGG(N_3_)CCGA-3′ was found in all component genes of this cluster in both *A. viridinutans* and *A. udagawae* (Figure 8C). The positions of the consensus sequence were highly conserved between the species. Collectively, *cis*-elements for the SM gene clusters with a C6-type transcription factor were well-conserved in an interspecies manner.

## Discussion

Comparative genomics studies regarding fungal SMs have been conducted intensively during the last decade, revealing the genetic diversity, universality, and plasticity of SM gene clusters in fungal genomes (Hansen et al., 2015; Nielsen et al., 2017; Vesth et al., 2018; Kjærbølling et al., 2020). Our genomic study provides a comprehensive catalog of SM backbone genes in *A. lentulus, A. udagawae, A. viridinutans, A. pseudoviridinutans, A. fumigatiaffinis, A. novofumigatus*, and *A. thermomutatus*, whereas those of *A. fumigatus* and *N. fischeri* have been well-investigated previously. Larsen et al. reported auranthine, cyclopiazonic acid, neosartorin, pyripyropene A, and terrein as major metabolites of *A. lentulus* (Larsen et al., 2007), which is supported by the presence of the corresponding backbone genes (*cpaA* for cyclopiazonic acid, *nsrB* for neosartorin, *pyr2* for pyripyropene A, and *terAB* for terrein) in the genome (Supplementary Table 5). Integrating studies of natural products and genomics will be a good way to further expand our understanding of fungal SM production.

*A. fumigatus* was estimated to have the largest number of characterized SM gene clusters among fungi even though the number of signature genes predicted by antiSMASH or other research was relatively small (~34 genes) (Sanchez et al., 2012; Mead et al., 2019). However, in comparison, the other species of *Aspergillus* section *Fumigati* possess 46–86 SM backbone genes. In the present study, we identified 10 SM backbone genes that are conserved in all the tested species of *Aspergillus* section *Fumigati*. Interestingly, the ASF-conserved SM gene clusters included the clusters responsible for the pathogenicity-related metabolites siderophores, DHN-melanin, and gliotoxin. This genomic signature indicated that these species have considerable potential for opportunistic pathogenicity, although some of the species have not been reported as pathogens. Transcriptome analysis of *N. fischeri, A. lentulus, A. udagawae*, and *A. viridinutans* as well as *A. fumigatus* revealed that expression profiles for the pathogenicity-related metabolite clusters were different among the species. For instance, the cluster for DHN-melanin was only activated in *A. fumigatus* and *A. viridinutans* on culture on PDA. This is consistent with what is known about spatiotemporal production of DHN-melanin. DHN-melanin is reported to be produced during the conidiation stage and exists on the surface of conidia in *A. fumigatus* (Chang et al., 2020). It is noteworthy that in the present study *A. fumigatus* and *A. viridinutans* produced conidia during culture on PDA, whereas conidiation was not observed for *N. fischeri, A. lentulus*, or *A udagawae*. If they were placed in conditions where conidia are produced, DHN-melanin may be biosynthesized and cover the conidial surface. Coordinate induced expression of the *gli* genes of *A. fumigatus, A. lentulus*, and *A. udagawae* was observed in certain conditions (Figure 5; Table 3). However, besides *A. fumigatus*, none of the species of *Aspergillus* section *Fumigati* tested were able to synthesize gliotoxin in CD culture (Supplementary Figure 2). It is possible that medium-dependent post-transcriptional regulation affects the production of fungal metabolites. Notably, *N. fischeri* was recently reported to produce gliotoxin when grown on CD agar or 5% blood agar plates at 37°C for 4 d (Knowles et al., 2020). This finding also supported the hypothesis that widely conserved BGCs tend to be more active in each species.

One of the important results presented here is that the ASF-conserved SM backbone genes were transcriptionally more active than species-unique genes in species of *Aspergillus* section *Fumigati* (Figure 4B). This tendency was also observed among all genes. Only a small fraction (3.5–6.7%) of ASF-conserved genes were silent, whereas the proportion of unexpressed species-unique genes was 37–56% (Figure 4A). Accordingly, the magnitude of expressed species-unique genes was lower than that of ASF-conserved genes (Figure 3). This significant difference can be explained by the presence of genes related to housekeeping machinery and primary metabolism among the ASF-conserved genes, which are in general kept highly activated throughout cell growth. With regard to secondary metabolism, the narrowly distributed SM genes, in particular the species-unique SM genes, tended to be less preferentially expressed. During the course of evolution, transcriptional regulation may be a potential target for the degeneration of secondary metabolism, although further clarification is required.

Transcriptome analysis of five species in four different conditions allowed us to comprehensively explore conditions for activation of SM gene clusters. For example, the backbone genes for DHN-melanin and trypacidin were highly expressed in *A. fumigatus* when grown on PDA (Supplementary Table 3). In *N. fischeri*, the NRPS genes for fusarinine C and triacetylfusarinine C were highly expressed in PDB (Supplementary Table 4). In *A. lentulus*, the backbone genes for fumarylalanine and terrein were highly expressed in PDB (Supplementary Table 5). The genes for fumarylalanine and hexadehydroastechrome were markedly expressed in SB and on PDA, respectively in *A. udagawae*, whereas genes for fumigaclavine C, neosartoricin/fumicycline A, and viriditoxin showed high expression levels in *A. viridinutans* in preferential conditions (Supplementary Tables 6, 7). Notably, fumicycline A production is induced in *A. fumigatus* when it is cocultured with *Streptomyces rapamycinicus* (König et al., 2013). Given that *A. viridinutans* produces this metabolite in monoculture, the molecular mechanisms underlying the transcriptional activation are likely to be different between the two species. Viriditoxin production was previously reported in *A. viridinutans* as well as *Paecilomyces variotii* (Urquhart et al., 2019). This metabolite shows a variety of biological activities such as potent inhibition of the bacterial cell division-related protein FtsZ (Wang et al., 2003), suggesting a protective role in competition against other microorganisms. Biosynthesis genes for terrein were identified in *A. terreus*, and terrein was produced in PDB and on PDA (Zaehle et al., 2014). Intriguingly, we showed that *A. lentulus* also produced large amounts of terrein in these conditions (Figure 6C). The consistency in the conditions required for terrein production suggests that the *ter* cluster is regulated in a similar manner in *A. lentulus* and *A. terreus*. This in turn suggests that the fungal SM gene clusters retain their transcriptional regulatory mechanism in different hosts.

Coordinated expression of the genes in SM BGCs involves a cluster-specific transcription factor or/and epigenetic regulator. Some BGCs contains transcription factors that play a pivotal role in activation of the cluster. The *gli, ter*, and *vdt* clusters have been investigated in *A. fumigatus, A. terreus*, and *P. variotii*, respectively. The transcription factors (GliZ, TerR, and VdtR) of each cluster were demonstrated to play an essential role in activation of the cluster and metabolite production (Schoberle et al., 2014; Gressler et al., 2015; Urquhart et al., 2019). Comparative studies here showed that the consensus transcription factor binding sequences for the genes in the BGCs are highly conserved among different species. However, different expression patterns of the BGCs were observed in different species, resulting in different metabolite production (e.g., of gliotoxin) (Supplementary Figure 2). One explanation for this inconsistency is that variations in the sequences of the promoter regions excepting the consensus sequences might affect the efficiency of RNA polymerase binding. Alternatively, other regulators might participate in regulating the expression of the clusters, which could lead to changes in the environmental cues required for cluster activation and metabolite production. As a consequence, transcriptional regulation of secondary metabolism might be diversified in the course of adaptation to host-specific environments.

In conclusion, we combined comparative genomic and transcriptomic analyses to study variations in the transcriptional activities of fungal BGCs in closely related species. This research provides a perspective on how the BGC distribution may be correlated with the tendency for transcriptomic silent SM-related genes. On the basis of our findings, diversification of transcriptional regulation might occur in the course of the evolution and degeneration of SM gene clusters. Further efforts to characterize such transcriptional diversity will expand our understanding of the evolutionary processes affecting fungal secondary metabolism.

## Materials and Methods

### Fungal Strains

The strains *A. fumigatus* Af293, *A. lentulus* IFM 54703, *A. udagawae* IFM 46973, *A. viridinutans* IFM 47045, *A. pseudoviridinutans* IFM 55266, and *N. fischeri* NRRL 181 were provided through the National Bio-Resource Project, Japan (http://www.nbrp.jp/) and are preserved at the Medical Mycology Research Center, Chiba University. The genomes of *A. lentulus* IFM 54703 (Kusuya et al., 2016) and *A. udagawae* IFM 46973 (Kusuya et al., 2015) were previously sequenced, and the data were retrieved from the NCBI database. *A. viridinutans* strain IFM 47045 (NRRL 4365) was isolated from rabbit dung in Australia. *A. pseudoviridinutans* strain IFM 55266 was isolated from a patient in Japan and identified using tubulin and calmodulin partial gene sequences (Lyskova et al., 2018).

### Culture Conditions

Strains were grown in liquid PDB (BD Difco, Franklin Lakes, NJ, USA), SB (BD Difco), or CD (BD Difco) at 37°C for 5 d by inoculating each culture with three 0.5-cm^2^ agar plugs. For asexual stage culture, mycelia cultured in PDB at 37°C for 3 d were harvested using a Miracloth, washed with distilled water, and then placed onto PDA plates (BD Difco) for another 2 d of culture at 37°C.

### Genome Sequencing

The genomic DNAs of *A. viridinutans* and *A. pseudoviridinutans* were extracted from a 2-d-old culture using phenol-chloroform and NucleoBond buffer set III (TaKaRa, Shiga, Japan). The DNA was fragmented in an S2 sonicator (Covaris, MA, USA), and then purified using a QIAquick gel extraction kit (Qiagen, CA, USA). A paired-end library was constructed using an NEBNext Ultra DNA Library Prep Kit (New England BioLabs, MA, USA) and NEBNext Multiplex Oligos (New England BioLabs) in accordance with the manufacturer's instructions. Mate-paired libraries with insert sizes of 3.5–4.5, 5–7, and 8–11 kb were generated using the gel selection-based protocol of the Nextera Mate Pair Kit (Illumina, San Diego, CA, USA) and a 0.6% agarose gel, in accordance with the manufacturer's instructions. The quality of the libraries was determined using an Agilent 2100 Bioanalyzer (Agilent Technologies, Santa Clara, CA, USA). Paired-end sequencing (100 bp) was performed by HiSeq 1500 (Illumina) using HiSeq reagent kit v1, in accordance with the manufacturer's instructions; 150-bp paired-end sequencing on a HiSeq X system (Illumina) was carried out by GENEWIZ (South Plainfield, NJ, USA).

### Genome Assembly and Gene Prediction

Adapters and low-quality bases from Illumina reads were trimmed by Trim Galore (ver. 0.6.4) (Krueger, 2015) with default settings (http://www.bioinformatics.babraham.ac.uk/projects/trim_galore/). Mitochondrial genomes were assembled using GetOrganelle (ver. 1.6.4) (Jin et al., 2020) from the trimmed reads. To filter the mitochondrial reads, the trimmed reads were aligned against mitochondrial genomes by BWA (ver. 0.7.17-r1188) (Li and Durbin, 2009), and the mapped reads were filtered by SAMtools (ver. 1.9) (Li et al., 2009) and SeqKit (Shen et al., 2016). The assembly of nuclear genomes of *A. udagawae, A. viridinutans*, and *A. pseudoviridinutans* was carried out by ALLPATHS-LG (ver. R52488) (Gnerre et al., 2011).

The annotation of assembled genomes of *A. lentulus, A. udagawae, A. viridinutans*, and *A. pseudoviridinutans* was performed by Funannotate pipeline (ver. 1.7.4) (https://funannotate.readthedocs.io/en/latest/). Following identification of repeat sequences by RepeatModeler (ver. 1.0.11) (http://www.repeatmasker.org/RepeatModeler.html) and RepeatMasker (ver. 4.0.7) (https://www.repeatmasker.org), Funannotate *ab initio* prediction was performed with the option “–busco_seed_species=aspergillus_fumigatus” by Augustus (ver. 3.3.3) (Stanke et al., 2006), GeneMark-ES (ver. 4.38) (Ter-Hovhannisyan et al., 2008), GlimmerHMM (ver. 3.0.4) (Majoros et al., 2004), and SNAP (ver. 2006-07-28) (Ian, 2004) using exon hints from the proteins of *A. fumigatus* Af293 and *N. fischeri* NRRL 181 downloaded from the *Aspergillus* Genome Database (Cerqueira et al., 2014). Functional annotation of predicted genes was performed by using the SwissPROT (Bairoch and Apweiler, 2000), InterPro (ver. 5.42-78.0) (Jones et al., 2014; Mitchell et al., 2019), eggNOG (ver. 4.5.1) (Buchfink et al., 2015; Huerta-Cepas et al., 2016), MEROPS (ver. 12.0) (Rawlings et al., 2018), and dbCAN (ver. 8.0) (Yin et al., 2012) databases. SM gene clusters were predicted by antiSMASH (ver. 4.2.0) (Blin et al., 2019). The completeness of draft genomes and predicted proteins was evaluated by BUSCO (ver. 4.0.6) (Seppey et al., 2019) with the database eurotiales_odb10. Genome synteny analysis between *A. fumigatus* and other species was conducted per Kjærbølling et al. (2020). Most tools were obtained through Bioconda (Grüning et al., 2018).

### Molecular Phylogenetic Analysis

A phylogenetic tree of *A. fumigatus* and related species was constructed using orthologs common in 11 species including *A. clavatus* NRRL 1 (Fedorova et al., 2008) and *A. nidulans* FGSC A4 (Galagan et al., 2005) as the outgroup. Orthologous genes among the 11 species were identified by OrthoFinder (ver. 2.3.12) (Emms and Kelly, 2019). Then, 4,465 single copy ortholog genes were aligned by using MAFFT (ver. 7.471) (Katoh and Standley, 2013), and well-aligned regions were extracted using Gblocks (ver. 0.91b) (Talavera and Castresana, 2007) with the options “-t=p, -b4=5, -b5=h.” A total of 4,465 aligned sequences of each strain were concentrated into one long protein sequence. A phylogenetic tree was constructed using multithreaded RAxML (ver. 8.2.12) (Stamatakis, 2014), the PROTGAMMAWAG model, and 100 bootstrap replicates, and visualized by iTOL (Letunic and Bork, 2019).

### Identification of SM Backbone Genes

Identification of SM genes was performed with antiSMASH (Blin et al., 2019). NRPS, PKS, and PKS-NRPS hybrid genes were extracted from the results. The orthologous relationships were determined by RBH with the criteria of BLASTp (ver. 2.7.1+) coverage >80% and identity >80% (Camacho et al., 2009). A cladogram was constructed based on a binary matrix (presence/absence of the PKSs and NRPSs) using Cluster 3.0 (http://bonsai.hgc.jp/~mdehoon/software/cluster/software.htm#ctv). A tree and heat map were constructed using Tree View (Saldanha, 2004).

### RNA Sequencing (RNA-Seq) and Data Analysis

Each strain was cultured, harvested, and cells were ground into a fine powder using a mortar. Total RNA was isolated using Sepazol-RNA Super G (Nacalai, Kyoto, Japan) in accordance with the manufacturer's instructions. The RNA isolation was carried out with two biological replicates, and equivalent quantities of the total RNA were pooled for preparation of the RNA-Seq libraries. The RNA-Seq libraries were constructed using a KAPA mRNA Hyper Prep Kit (Nippon Genetics, Tokyo, Japan), in which mRNA was purified by poly-A selection, second-strand cDNA was synthesized from the mRNA, the cDNA ends were blunted and polyAs added at the 3′- ends, and appropriate indexes were ligated to the ends. The libraries were PCR amplified, and the quantity and quality were assessed using a Bioanalyzer (Agilent Technologies). Each pooled library was sequenced using Illumina HiSeq 1500 apparatus. The gene expression levels were estimated using a previously described method (Takahashi et al., 2017). Briefly, the sequencing reads were mapped to the reference genomes using STAR (ver. 2.4.2a) (Dobin et al., 2013). A raw read count was conducted using HTSeq (ver. 0.5.3p3) (Anders et al., 2015), and transcript abundances were estimated as TPMs (Conesa et al., 2016). The expressed PKS and NRPS genes were identified using the 5% mean TPM criterion.

### MIDDAS-M Analysis

Binary logarithms of TPM values generated in the four culture conditions were analyzed using the MIDDAS-M algorithm to detect gene clusters in which certain condition(s) coordinately expressed or repressed genes (Umemura et al., 2013). Briefly, the induction ratio of each gene was evaluated for every pairwise combination of the four culture conditions. After *Z*-score normalization, the gene cluster expression scores were calculated for each gene using the algorithm. The maximum cluster size was set as 30. The threshold to detect clusters was set to the value corresponding to a false positive rate of 0, which was evaluated from data in which the original gene order was randomly shuffled. The threshold values were 2.1E5, 2.3E5, 2.4E5, 1.3E5, and 3.5E5 in *A. fumigatus, A. lentulus, A. udagawae, A. viridinutans*, and *N. fischeri*, respectively.

### Extraction of Compounds and High-Performance Liquid Chromatography (HPLC) Analysis

For gliotoxin detection, 5 mL of culture supernatant was extracted with an equivalent volume of ethyl acetate. The organic layer was concentrated *in vacuo*, and the precipitate was dissolved in 2 mL of dimethylsulfoxide (DMSO). The DMSO solution (5 μL) was subjected to HPLC analysis, which was performed using an Infinity1260 modular system (Agilent Technologies, Santa Clara, CA) consisting of an autosampler, high-pressure pumps, a column oven, and a photodiode-array detector with InfinityLab Poroshell 120 EC-C18 column (particle size: 2.7 μM; length: 100 mm; internal diameter: 3.0 mm; Agilent Technologies). Running conditions were gradient elution, 5–40% acetonitrile in water over 18 min; flow rate, 0.8 mL min^−1^; and detection at 254 nm. Gliotoxin production was identified by comparing the retention times and the UV spectra of natural products with those of a gliotoxin standard that was purchased from Sigma-Aldrich (St. Louis, MO).

For terrein detection, 5 μL of culture supernatant was subjected to HPLC analysis, which was performed using the same HPLC system as described above. Running conditions were gradient elution, 5–100% acetonitrile in water over 30 min; flow rate, 0.8 mL min^−1^; and detection at 254 nm. Terrein production was identified in the same manner as gliotoxin production. The authentic standard was purchased from Cayman Chemical Company (Ann Arbor, MI).

### *Cis*-Element Detection

Sequence 500 bp upstream of each gene encoded in *gli* and *vdt* clusters was retrieved as the promoter sequence. Two-block motifs were sought by BioProspector (Release 2) (Liu et al., 2001) with the options “-d 1 -W 4 -w 4 -g 3 -G 3.” The sequence logos were depicted by WebLogo (Crooks et al., 2004).

## Data Availability Statement

The whole-genome sequences of *A. viridinutans* IFM 47045 and *A. pseudoviridinutans* IFM 55266 have been deposited at DDBJ/EMBL/GenBank under accession numbers BOPL01000001-47 and BHVY01000001-24, respectively. Re-assembled sequence data of *A. lentulus* IFM 54703 and *A. udagawae* IFM 47045 have been also placed under accession numbers BCLY01000001, BCLY01000004-BCLY01000009, BCLY01000011-BCLY01000013, and BCLY01000016-BCLY01000017, and BBXM02000001-17, respectively. The raw RNA-Seq data from this work have been submitted to the DDBJ Short Read Archive under accession number PRJDB7496.

## Author Contributions

HT, MU, and DH designed the research. HT, MU, AN, MS, YK, S-iU, TY, and DH performed experiments and analyzed data. HT, AW, KK, and TY contributed new materials/tools. HT, MU, AN, TY, and DH wrote the manuscript. All authors contributed to the article and approved the submitted version.

## Conflict of Interest

The authors declare that the research was conducted in the absence of any commercial or financial relationships that could be construed as a potential conflict of interest.

## Supplementary Material

Supplementary Figure 1 ASF-conserved BGCs of nine species of *Aspergillus* section *Fumigati*. The genes predicted to be cluster components are indicated with blue arrows, whereas those indicated with white arrows are outside the cluster. Orange arrows indicate SM backbone genes, and red arrows indicate transcription factors. Black arrows indicate genes that show identity <80% compared with the corresponding gene in *A. fumigatus*.

Supplementary Figure 2 Production of gliotoxin. The strains were cultivated in CD medium, and the culture extracts obtained using ethyl acetate were analyzed by HPLC. Gliotoxin (**1**) production was identified by reference to the standard compound (Std.). The corresponding peak from *A. fumigatus* is indicated by an arrow, and the UV spectrum is shown in the red box. *Afu, A. fumigatus; Nfi, N. fischeri; Ale, A. lentulus; Aud, A. udagawae; Avi, A. viridinutans*.

Supplementary Table 1 List of SM backbone genes in the nine *Aspergillus* section *Fumigati* species.

Supplementary Table 2 The ASF-conserved BGCs.

Supplementary Tables 3–7 The SM backbone genes with expression values (TPM) in *A. fumigatus, N. fischeri, A. lentulus, A. udagawae*, and *A. viridinutans*.

Supplementary Table 8 Coordinately expressed gene clusters identified by MIDDAS-M analysis.

Supplementary Table 9 Comparison of *t*e*r* cluster between *A. lentulus* and *A. terreus*.
