# Supplementary material for: Interspecies Genomic Variation and Transcriptional Activeness of Secondary Metabolism-Related Genes in Aspergillus Section Fumigati
Source: Front Fungal Biol. 2021 Apr 16;2:656751. doi: 10.3389/ffunb.2021.656751 (PMC10512231; doi:10.3389/ffunb.2021.656751)
Supplement: Supplementary Figure 1 — ASF-conserved BGCs of nine species of Aspergillus section Fumigati. The genes predicted to be cluster components are indicated with blue arrows, whereas those indicated with white arrows are outside the cluster. Orange arrows indicate SM backbone genes, and red arrows indicate transcription factors. Black arrows indicate genes that show identity <80% compared with the corresponding gene in A. fumigatus. [file Presentation_1.PPTX]

## Slide 1
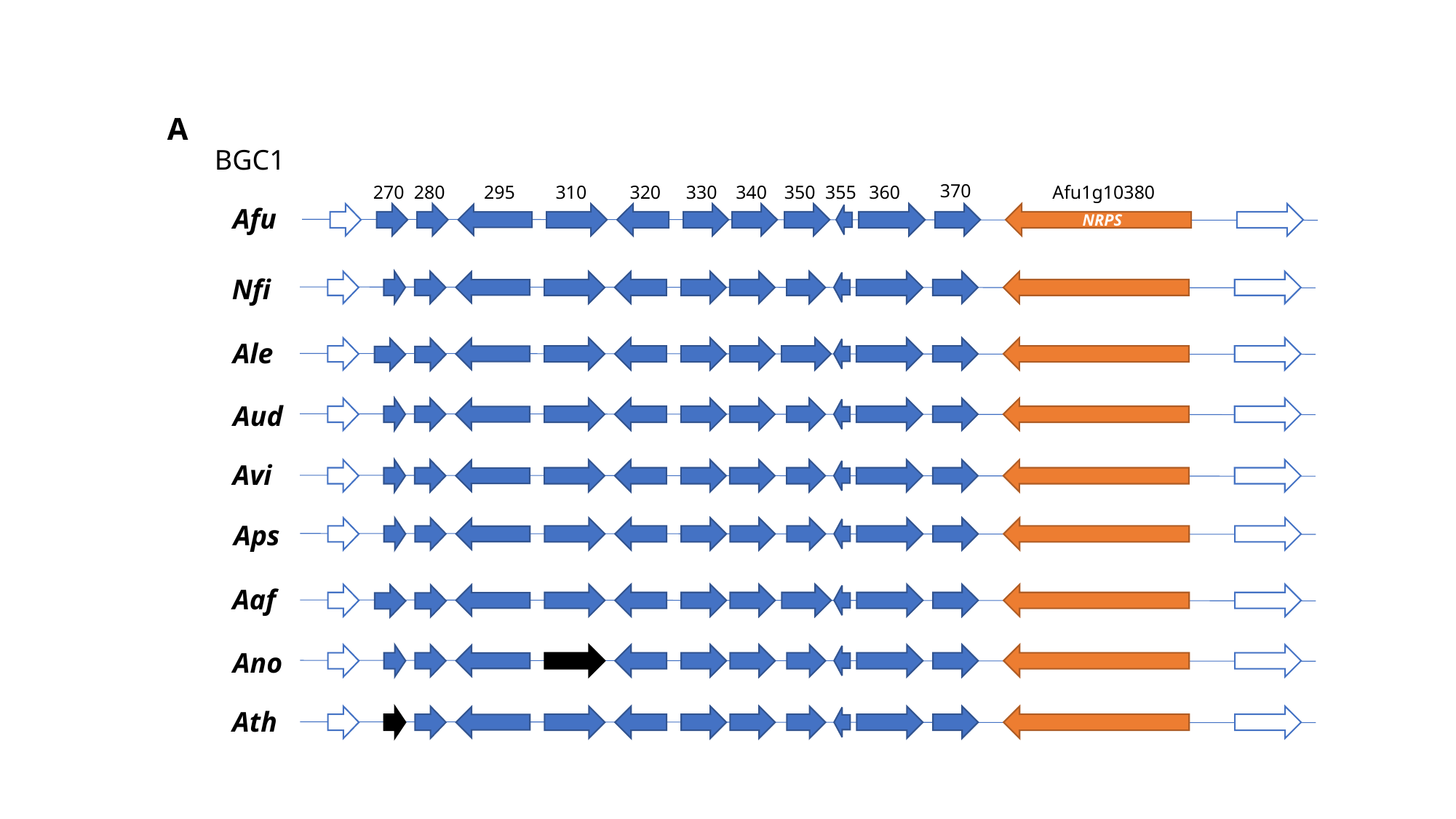

A
BGC1
370
270
280
295
310
320
330
340
350
355
360
Afu1g10380
Afu
NRPS
Nfi
Ale
Aud
Avi
Aps
Aaf
Ano
Ath

## Slide 2
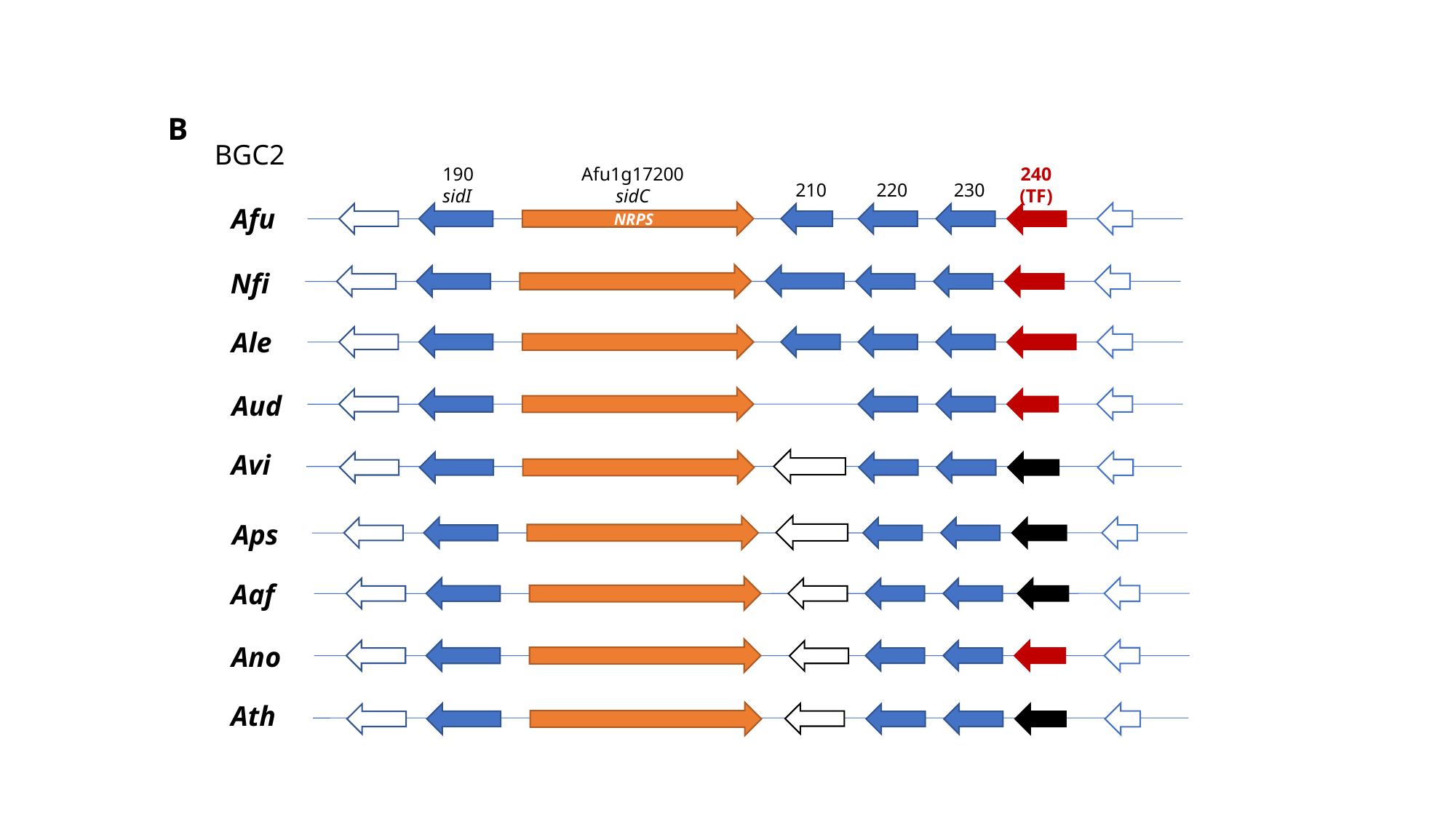

B
BGC2
190
sidI
240
(TF)
Afu1g17200
sidC
210
220
230
Afu
NRPS
Nfi
Ale
Aud
Avi
Aps
Aaf
Ano
Ath

## Slide 3
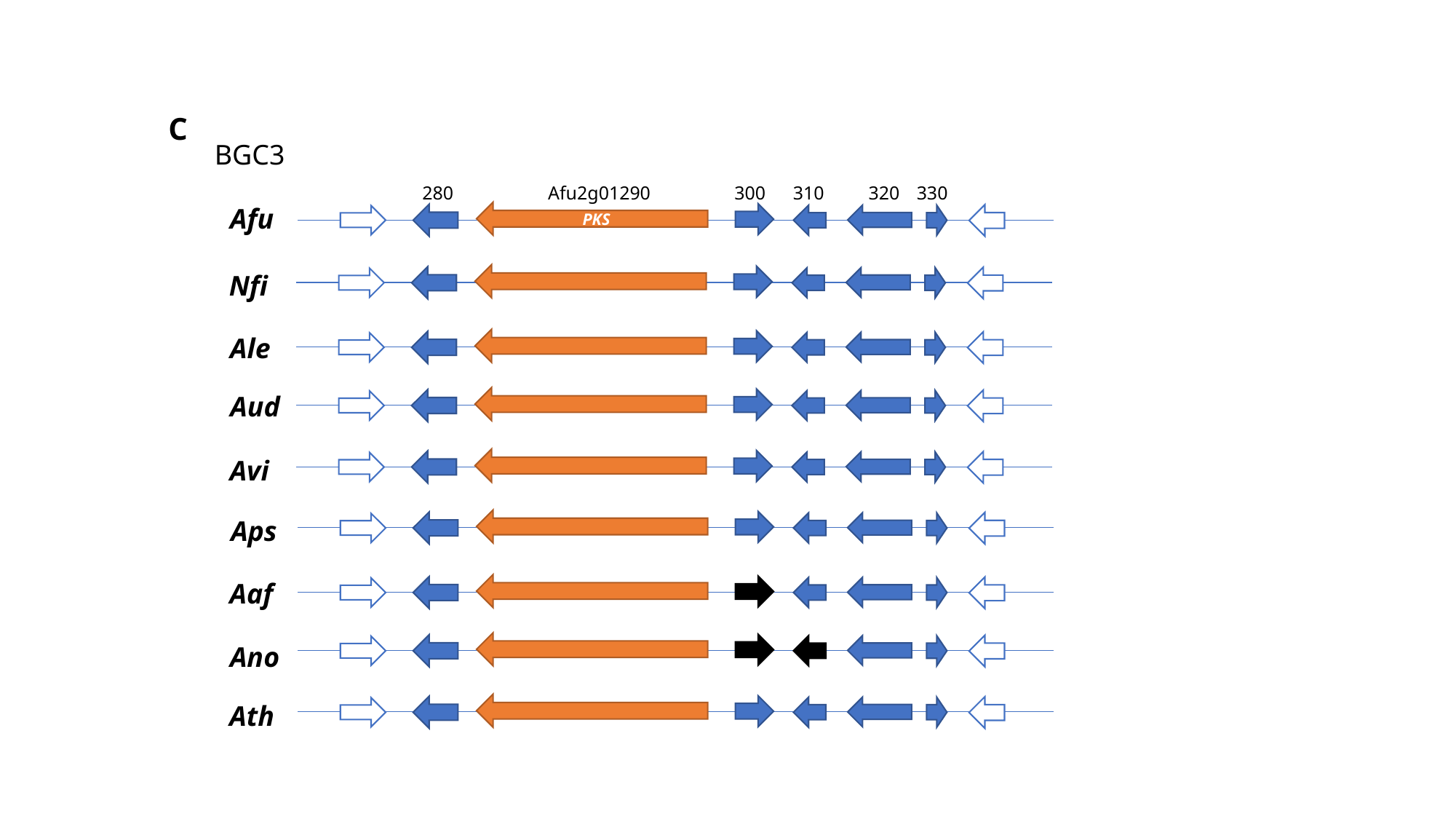

C
BGC3
280
Afu2g01290
300
310
320
330
Afu
PKS
Nfi
Ale
Aud
Avi
Aps
Aaf
Ano
Ath

## Slide 4
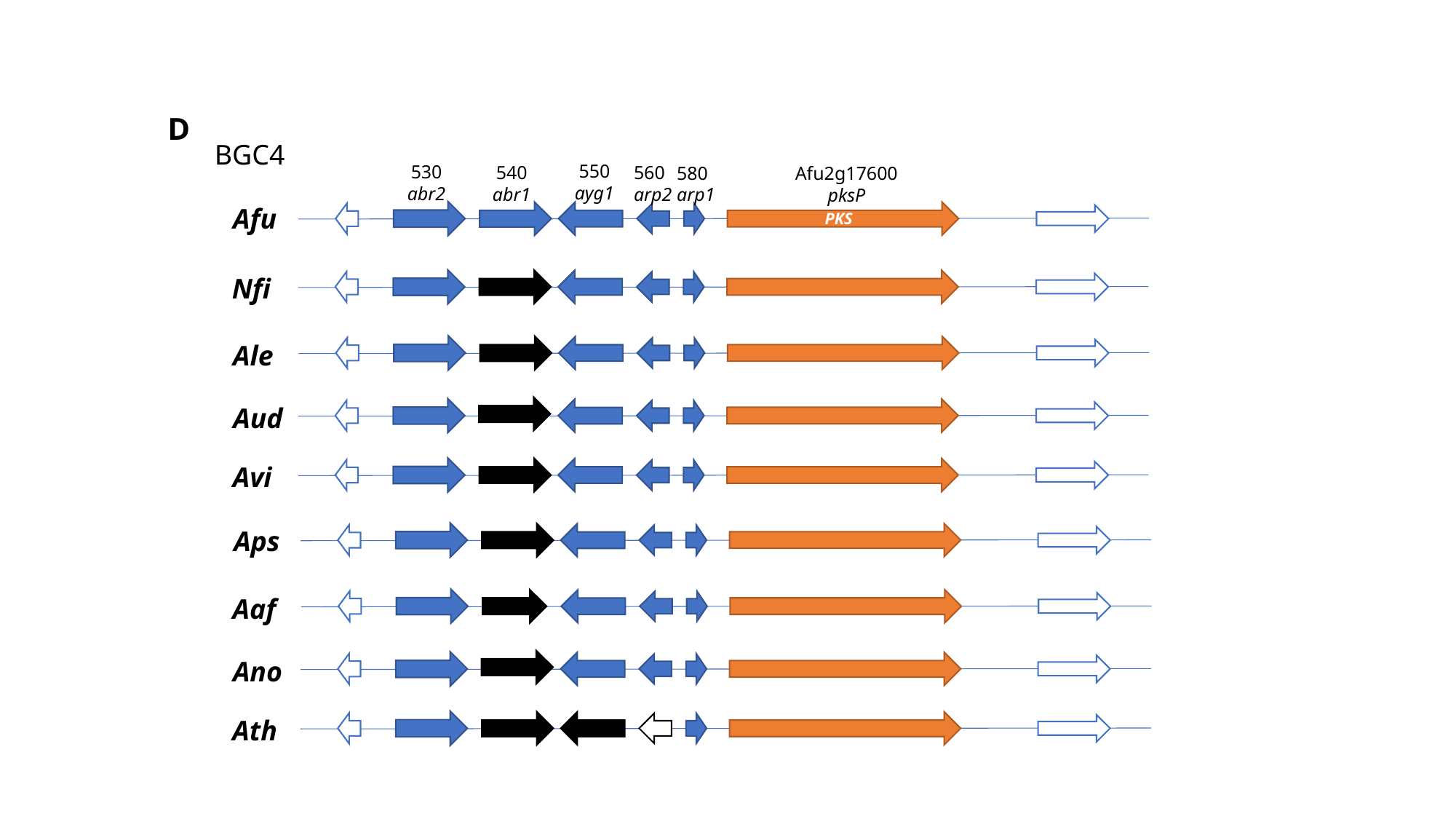

D
BGC4
550
ayg1
530
abr2
560
arp2
540
abr1
580
arp1
Afu2g17600
pksP
Afu
PKS
Nfi
Ale
Aud
Avi
Aps
Aaf
Ano
Ath

## Slide 5
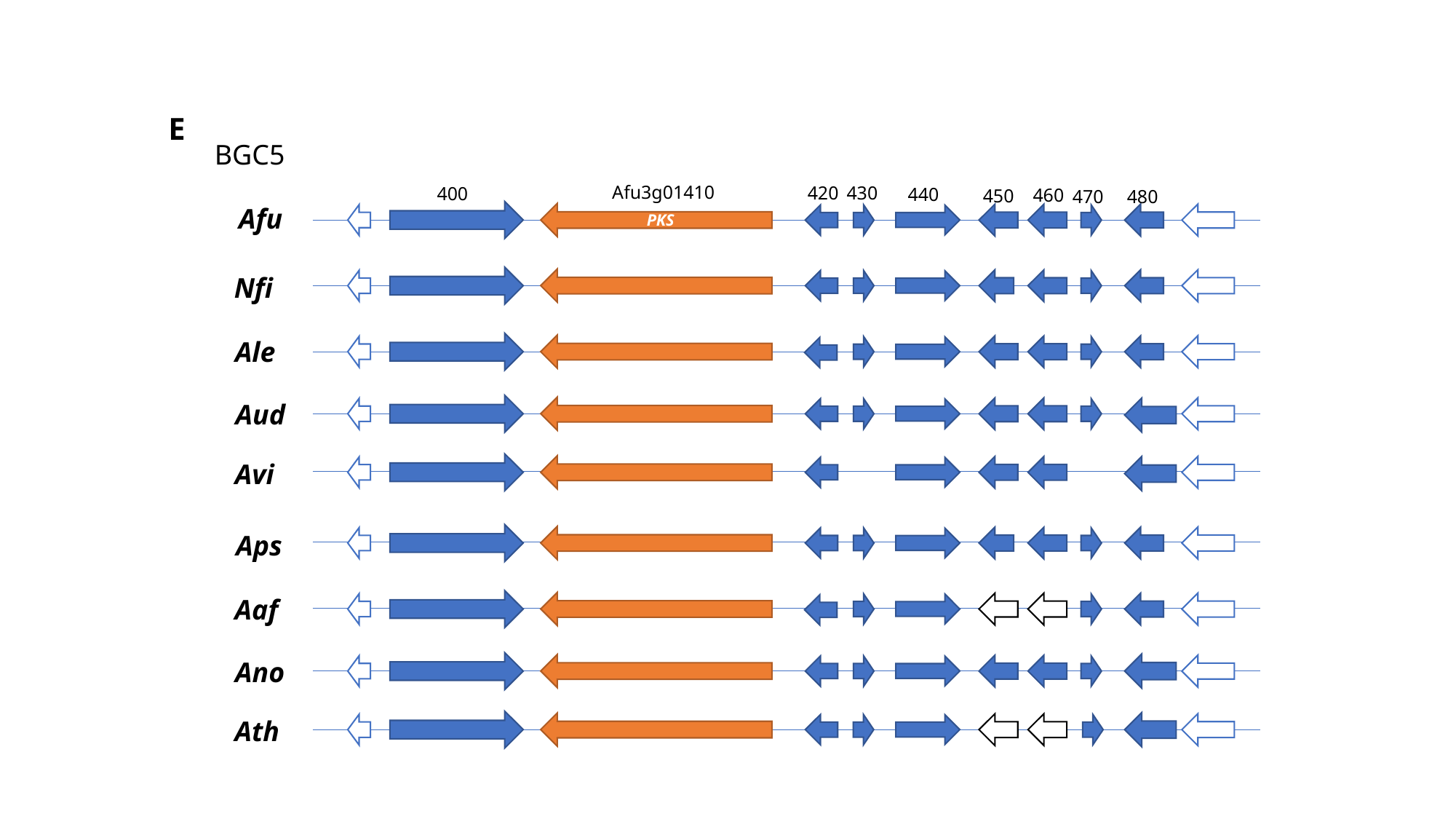

E
BGC5
Afu3g01410
430
420
400
440
460
450
480
470
Afu
PKS
Nfi
Ale
Aud
Avi
Aps
Aaf
Ano
Ath

## Slide 6
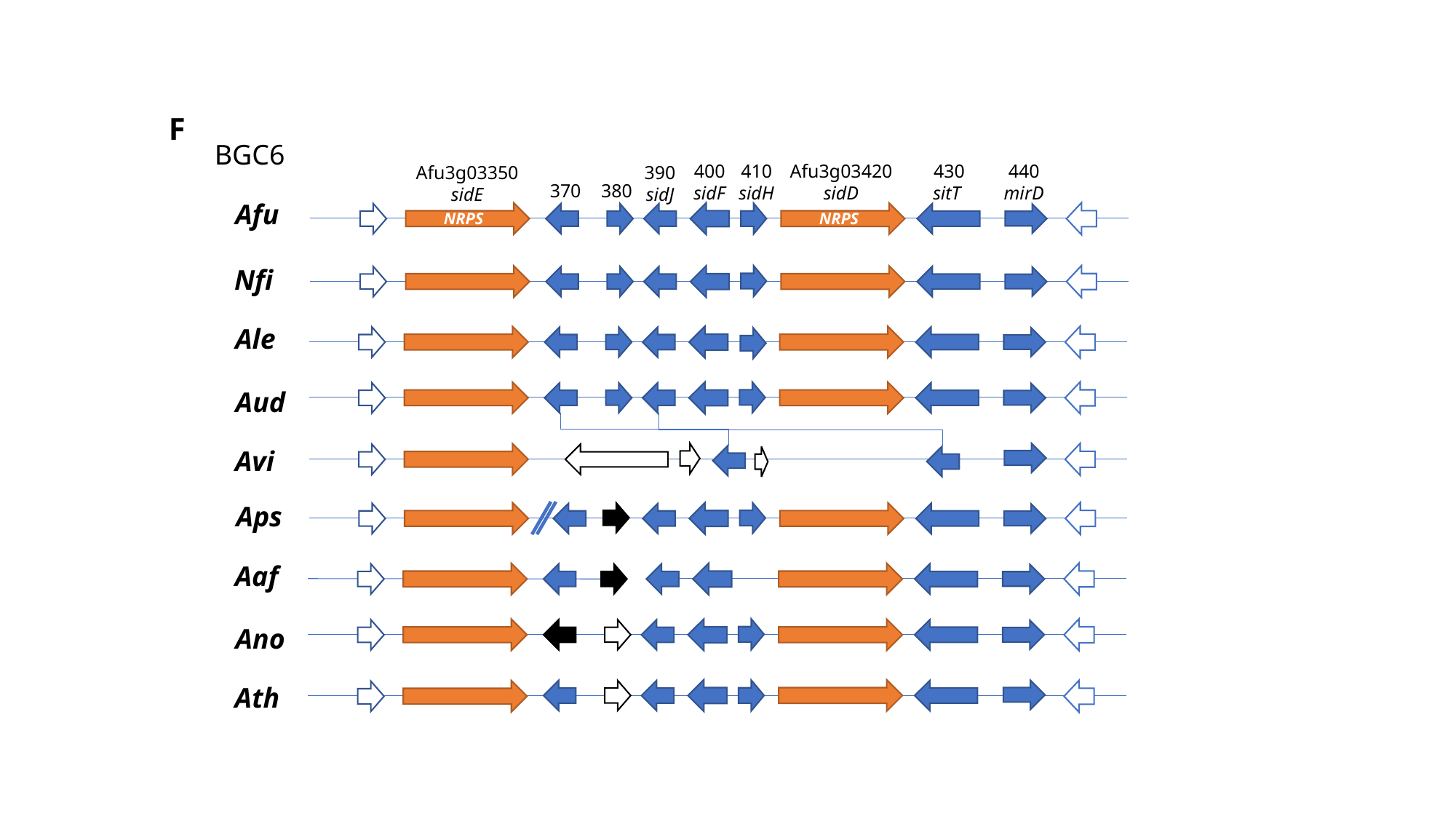

F
BGC6
Afu3g03420
sidD
430
sitT
400
sidF
410
sidH
440
mirD
390
sidJ
Afu3g03350
sidE
370
380
Afu
NRPS
NRPS
Nfi
Ale
Aud
Avi
Aps
Aaf
Ano
Ath

## Slide 7
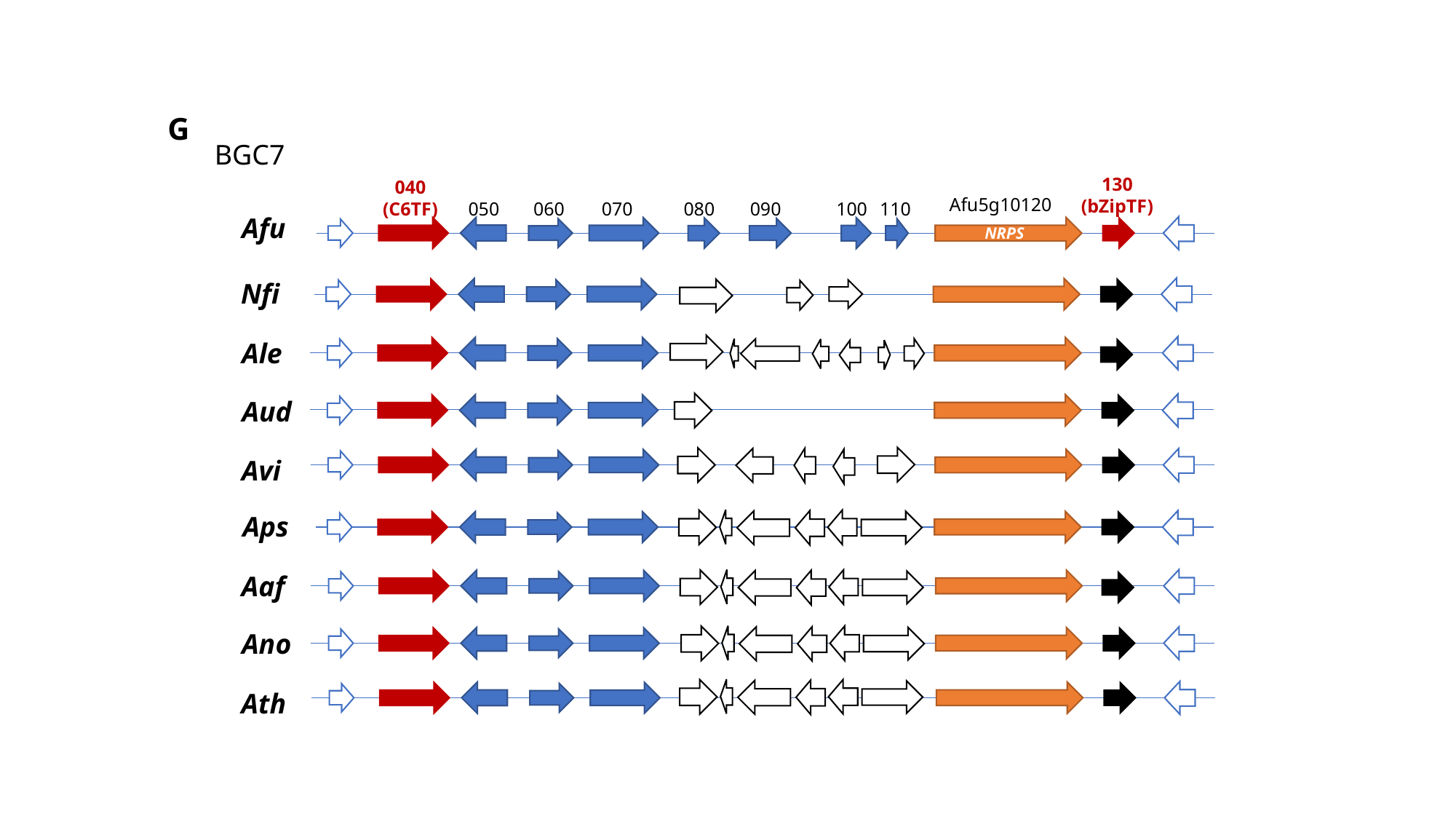

G
BGC7
130
(bZipTF)
040
(C6TF)
Afu5g10120
050
060
070
080
090
100
110
Afu
NRPS
Nfi
Ale
Aud
Avi
Aps
Aaf
Ano
Ath

## Slide 8
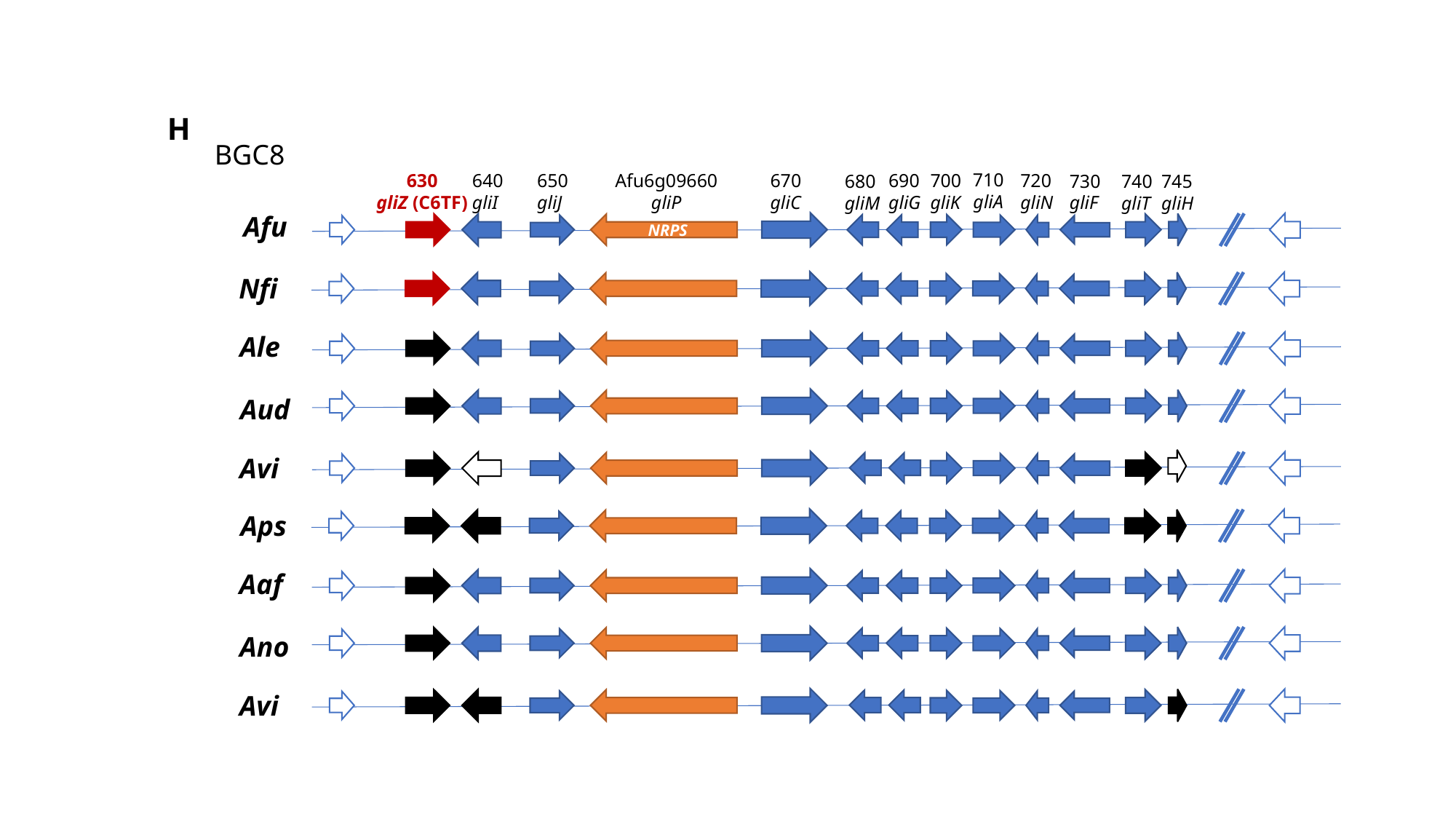

H
BGC8
710
gliA
650
gliJ
Afu6g09660
gliP
720
gliN
630
gliZ (C6TF)
640
gliI
670
gliC
690
gliG
700
gliK
730
gliF
680
gliM
740
gliT
745
gliH
Afu
NRPS
Nfi
Ale
Aud
Avi
Aps
Aaf
Ano
Avi

## Slide 9
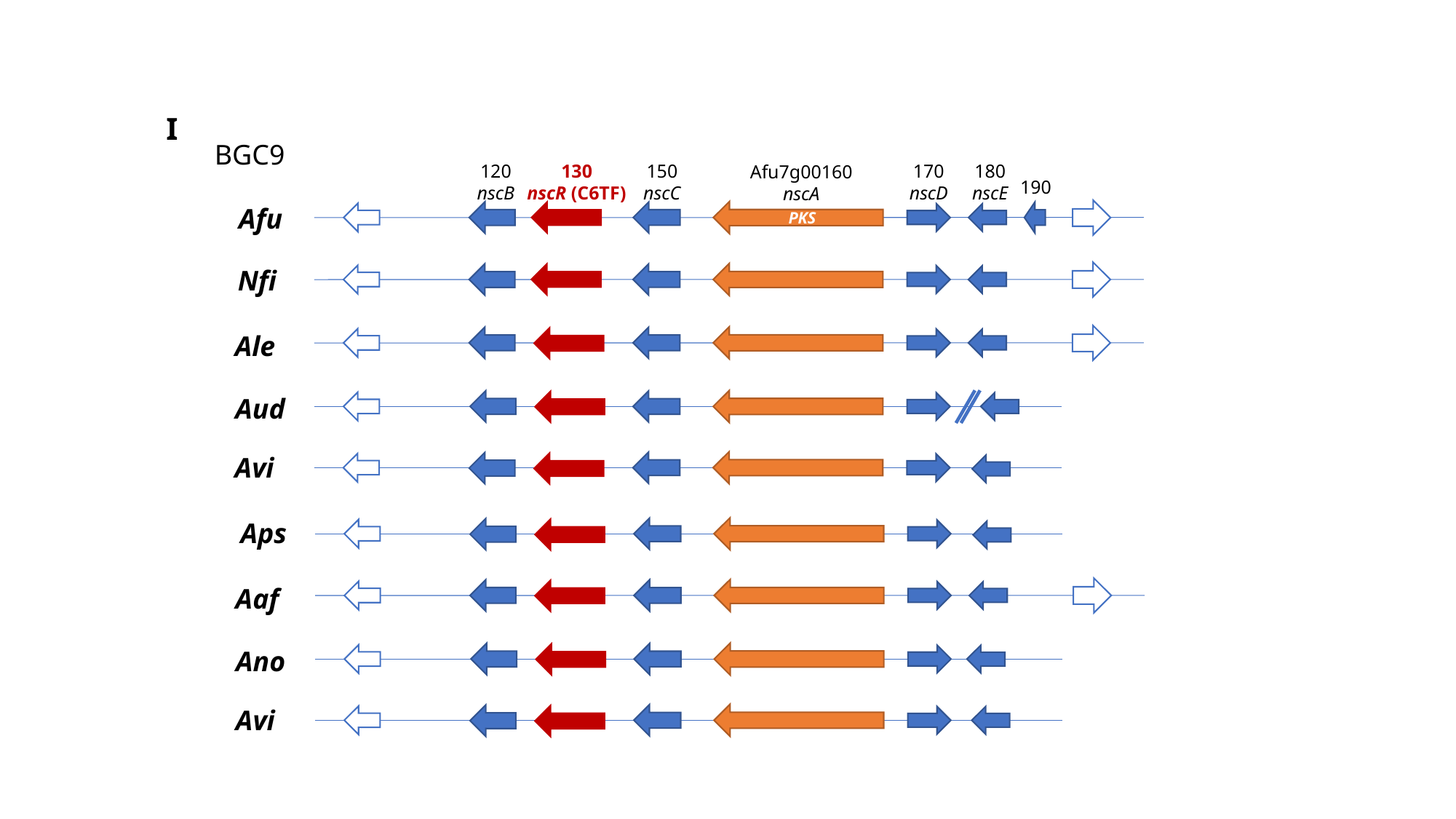

I
BGC9
130
nscR (C6TF)
170
nscD
150
nscC
120
nscB
180
nscE
Afu7g00160
nscA
190
Afu
PKS
Nfi
Ale
Aud
Avi
Aps
Aaf
Ano
Avi

## Slide 10
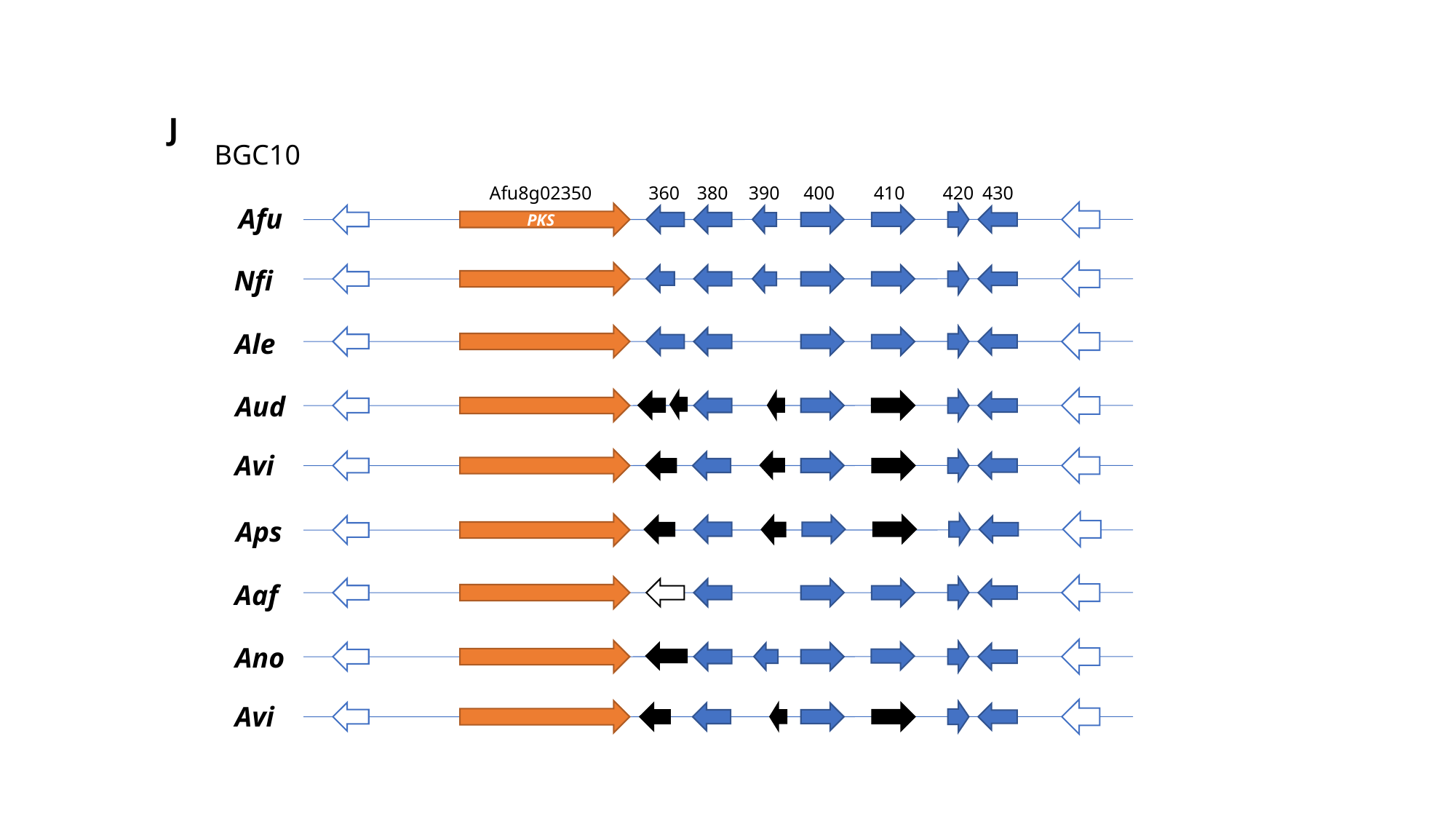

J
BGC10
Afu8g02350
360
380
390
400
410
420
430
Afu
PKS
Nfi
Ale
Aud
Avi
Aps
Aaf
Ano
Avi
