# Supplementary figures and images for: Interspecies Genomic Variation and Transcriptional Activeness of Secondary Metabolism-Related Genes in Aspergillus Section Fumigati
Source: Front Fungal Biol. 2021 Apr 16;2:656751. doi: 10.3389/ffunb.2021.656751 (PMC10512231; doi:10.3389/ffunb.2021.656751)

## Slide 1
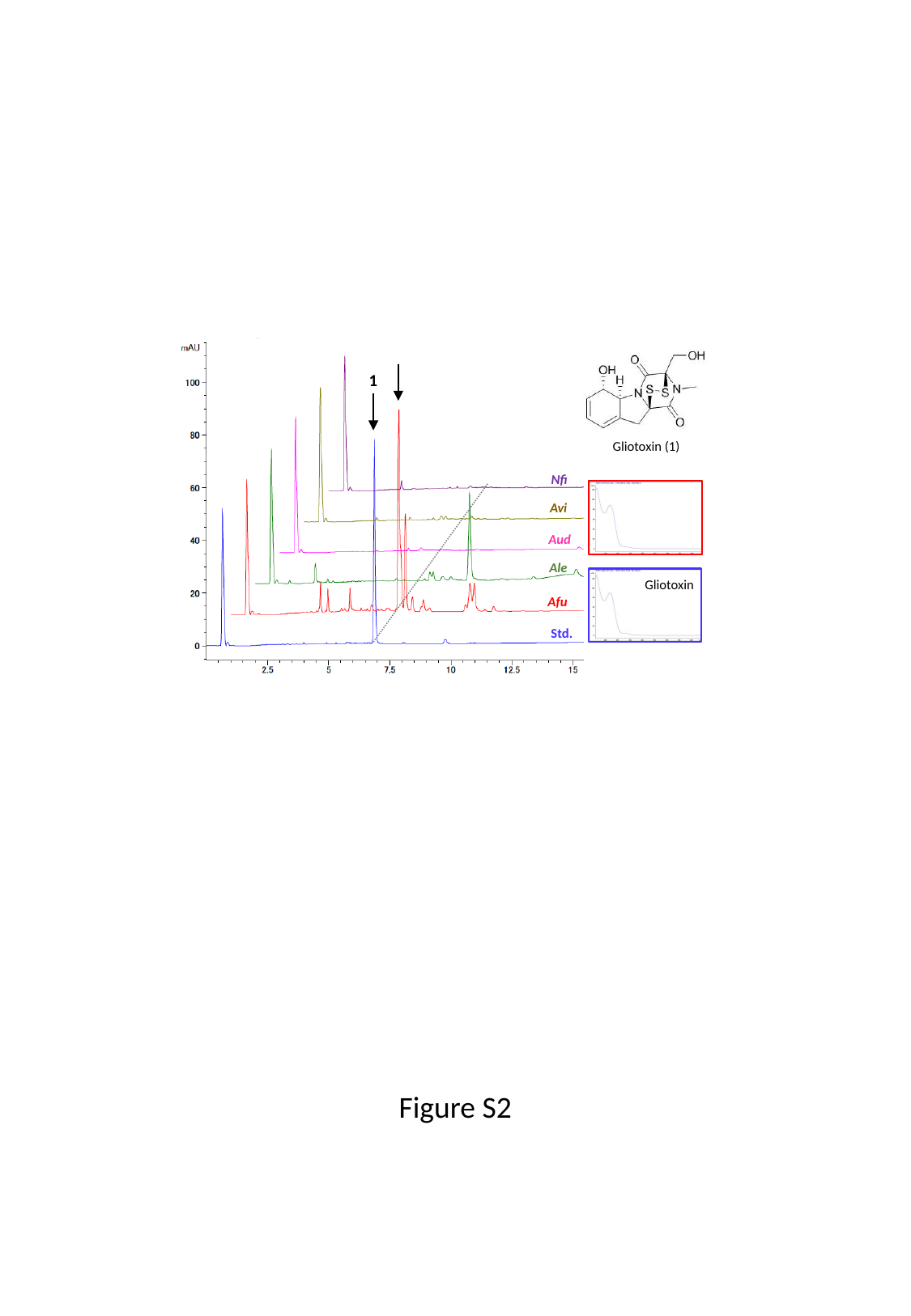

1
Gliotoxin (1)
Nfi
Avi
Aud
Ale
Gliotoxin
Afu
Std.
Figure S2

Supplement: Supplementary Figure 2 — Production of gliotoxin. The strains were cultivated in CD medium, and the culture extracts obtained using ethyl acetate were analyzed by HPLC. Gliotoxin (1) production was identified by reference to the standard compound (Std.). The corresponding peak from A. fumigatus is indicated by an arrow, and the UV spectrum is shown in the red box. Afu, A. fumigatus; Nfi, N. fischeri; Ale, A. lentulus; Aud, A. udagawae; Avi, A. viridinutans. [file Presentation_2.PPTX]
